# Melamine-Based Porous Organic Frameworks as Adsorbent Materials for the Removal of Organic Dyes from Wastewater

**DOI:** 10.3390/molecules31122022

**Published:** 2026-06-09

**Authors:** Salvatore Marullo, Giovanna Raia, Roberto Fiorenza, Martina Maria Calvino, Francesco Giannici, Giuliana Impellizzeri, Francesca D’Anna

**Affiliations:** 1 Dipartimento STEBICEF, Università degli Studi di Palermo, Viale delle Scienze Ed. 17, 90128 Palermo, Italy; giovanna.raia@unipa.it; 2 Dipartimento di Scienze Chimiche, Università degli Studi di Catania, Via A. Doria 6, 95125 Catania, Italy; roberto.fiorenza@unict.it; 3 Dipartimento DiFC, Università degli Studi di Palermo, Viale delle Scienze Ed. 17, 90128 Palermo, Italy; martinamaria.calvino@unipa.it (M.M.C.); francesco.giannici@unipa.it (F.G.); 4 CNR-IMM, Via Santa Sofia 64, 95123 Catania, Italy; giuliana.impellizzeri@ct.infn.it

**Keywords:** porous organic framework, organic dyes, adsorption, recycle

## Abstract

The pressing issues of organic pollutants contamination of aquatic ecosystems challenges current research. Herein, we prepared three melamine-based POFs, to remove organic dyes from water. Melamine was polymerized with 1,4-dibromobutane (POF-1,4), terephthalaldehyde (POF-TerA) and trimesic acid (POF-TriA), obtaining POFs of different structural order degree and aromaticity. POFs were characterized using FT-IR spectroscopy, thermal gravimetric analysis, BET, powder X-ray diffraction and scanning electron microscopy. They were employed to remove cationic (Rhodamine B, RhB and Methylene Blue, MB) and anionic dyes (Methyl Orange, MO and Eosin Yellow, EY), using UV-vis investigation. The adsorption process was studied from the kinetic and thermodynamic points of view and reusing the best adsorbent was also considered. Data collected evidence that adsorption capacity depends on the POF structure, with maximum adsorption capacity, according to Langmuir isotherm model, of 329 mg/g for POF-1,4/MO and 472 mg/g for POF-TerA/RhB. Interactions involved in the adsorption were also elucidated. Comparison with reported data demonstrates that our materials show comparable performance to some previously reported systems. Furthermore, POF-TriA, is effective for dye mixtures and reusable three times without performance loss, after washing with methanol, avoiding harsh acidic/basic treatments. Results obtained systematically relate the adsorption efficiency to structural features of melamine-based POFs, representing useful support in designing such materials to remove selected classes of contaminants.

## 1. Introduction

One of the most serious concerns of modern society is the contamination of water bodies that significantly reduces the amount of freshwater availability. Water is a natural resource for human well-being and ecosystem and, considering that earth provides less than 3% of freshwater, wastewater remediation plays a pivotal role.

Among the different contaminants, a significant share is made up by organic compounds, such as pharmaceutical active compounds, pesticides and synthetic organic dyes. In particular, organic dyes derive from the intense activity of textile industries. Global production amounts to more than 800,000 tons/year, but approximately 10–15% enters the ecosystem [1]. Their release in the environment is dangerous not only in terms of direct toxicity, but also upon degradation, as their by-products are known for risks of carcinogenesis and teratogenesis for humans.

During the years, the topic of wastewater treatment has generated a surge of interest and different approaches have been proposed by researchers, including catalytic degradation [2], membrane separation methods [3] and adsorption techniques [4,5]. The latter offer several advantages, like cost-effectiveness, possibility of reuse and recycle the adsorbent, broad application and low energy requirements. This explains why wastewater treatment has been performed using adsorbent of different nature, like supramolecular gels [6,7], clays [8], polymers [9], metal oxides [10], carbon nanotubes [11], graphene oxide [12] and so on.

It is generally recognized that good performance in adsorption processes is directly related to porosity and surface area of the adsorbent, together with the presence of definite adsorption sites. The above requirements have significantly boosted research, in the attempt to identify new porous materials that can be efficiently applied in such processes.

In this context, 2D nanomaterials, like MXenes [13,14], metal–organic frameworks [15] and covalent organic frameworks (COFs) [16] have been frequently claimed as valuable possibilities for the clean-up of most of pollutants in wastewater. COFs can be two-dimensional and three-dimensional and belong to the class of porous crystalline architectures [17,18,19]. Firstly, introduced by Yagi in 2005 [17], they possess a backbone structure made by light elements (B, C, N, O, etc.), connected by covalent bonds. Different linkages such as imine, hydrazone ketoenamine, imide spiroborate and so on have been investigated, allowing to tailor their structure for specific applications [20]. Differently from metal–organic frameworks and zeolites, they show unique architectures [21]. Furthermore, they have high thermal and chemical stability, together with low mass density endowing them with a large adsorption capacity. Thanks to their regular skeleton, tunable functionalities and versatile synthesis, these porous materials have been used in a plethora of areas, such as fluorescence detection [22], heterogeneous catalysis [23], energy storage [24], drug transport [25], environmental remediation [26,27,28] and chiral separation [29].

To date, there is no IUPAC definition of COFs, however in the literature this term is predominantly used for crystalline materials [30]. To avoid any confusion, we will use the term Porous Organic Framework (POF) for the non-crystalline counterpart of COFs, which beside crystallinity, maintain the other properties. This is consistent with the current terminological usage in the literature [31,32].

**Scheme 1:**
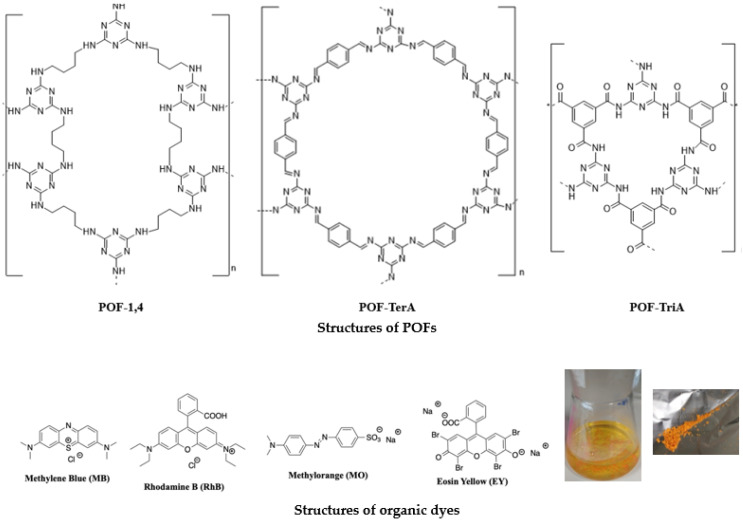
Structures of POFs and dyes used. Picture of an EY solution in contact with POF and POF after filtration (**bottom right**).

Bearing in mind all above considerations, in this work, we prepared three triazine-based POFs and carried out adsorption processes of organic dyes from wastewater. In particular, the main goal of the work was to evaluate how the different nature of the co-monomer, used in building the porous network, could affect the adsorption capacity of the materials. Under this light, melamine was polymerized in the presence of 1,4-dibromobutane, terephthalaldehyde and trimesic acid, obtaining adsorbents of different porosity, structural order and aliphatic/aromatic nature (Scheme 1).

After characterization of the porous network, using FT-IR, thermal gravimetric analysis (TGA), scanning electron microscopy (SEM) and BET, they were used for the removal of both cationic (Rhodamine B, RhB and Methylene Blue, MB) and anionic dyes (Methyl orange, MO and Eosin Yellow, EY) from wastewater. These materials conjugate synthetic accessibility with functional group diversity, allowing the correlation of their performance with the monomer structure. Although such materials were previously reported in the literature, to the best of our knowledge, they have not been used as sorbents for dyes removal.

Adsorption processes were investigated carrying out kinetic measurements, determining adsorption isotherms and investigating the interactions involved in the adsorption process. The effect of important operational parameters, like temperature, p*H* and ionic strength was also evaluated. Performance of the best adsorbent was also analyzed in the removal of dyes mixtures, and the possibility of regenerating and reusing it was also taken into consideration.

Collected results shed light on the relevance of the nature of the monomer used in determining the structural parameters of POFs, like porosity and aromaticity, that in turn affect their adsorption capacity and affinity to cationic or anionic dyes. Interestingly, the best material can be applied in the clean-up of the dye mixtures without loss in performance, also showing the possibility to be reused for at least three cycles.

## 2. Results

### 2.1. POFs Characterization

**Figure 1:**
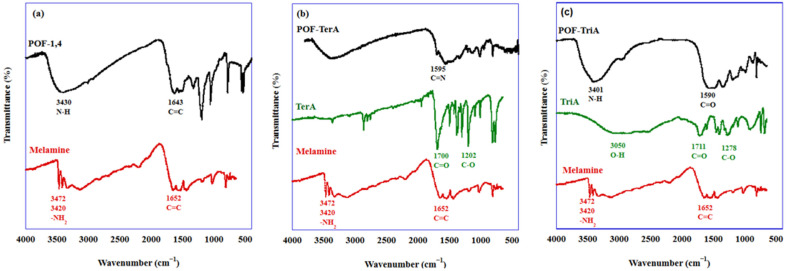
FT-IR spectra of POFs used.

POFs were synthesized using previously reported procedures [33,34,35], as detailed in Section 3 and Supplementary Information. In all cases, they were obtained with yields ranging from 62% to 75%, as colored powders. The materials obtained were first characterized, recording FT-IR spectra from KBr pellets. Results obtained are reported in Figure 1, together with spectra of precursors, to better evidence changes in the structure and identify signals corresponding to the formation of the bonds in the porous structure.

In the case of POF-1,4, the comparison between IR spectra of melamine and corresponding POF evidenced the disappearance of signals located at 3472, and 3420 cm^−1^, corresponding to the stretching of NH_2_ groups, replaced by a wider band centered at 3430 cm^−1^, deriving from the N-H group in the material (Figure 1a). On the other hand, in the case of POF-TerA, IR spectrum did not show the stretching bands located at 2870 and 1700 cm^−1^, corresponding to the aldehyde group, and the ones corresponding to the amino groups (3472 and 3420 cm^−1^). However, it was possible to identify the stretching band of the imine bond located at 1595 cm^−1^ which is consistent with previous reports in the literature concerning highly conjugated C=N bonds (Figure 1b) [36]. Finally, in the case of POF-TriA, (Figure 1c) IR spectrum of porous polymer did not exhibit stretching bands of the primary amino group of melamine, which is replaced by a single band at 3401 cm^−1^ corresponding to the N-H stretching of the amide group, associated with the appearance of the amide C=O stretching band at 1590 cm^−1^ in agreement with previous preparations reported in the literature [35].

Thermal stability of the materials, as obtained from TGA investigation, clearly indicates that the nature of the second component significantly affects their physico-chemical properties. For all samples, thermograms and DTGA traces are reported in Figure S1, in all cases, the temperature corresponding to the first degradation step (*T_d_*_1_) was considered as the operational limit for the corresponding POF.

In Table 1, decomposition temperatures, obtained from DTGA traces, as a function of the different nature of material, are reported.

Analysis of *T_d_*_1_ values reported in the Table allows identifying the POF-TriA as the most stable one. Indeed, the first decomposition process was detected at 360 °C, whereas in the other cases decomposition temperature gradually decreases from 250 °C down to 127 °C, going from POF-1,4 to POF-TerA.

**Figure 2:**
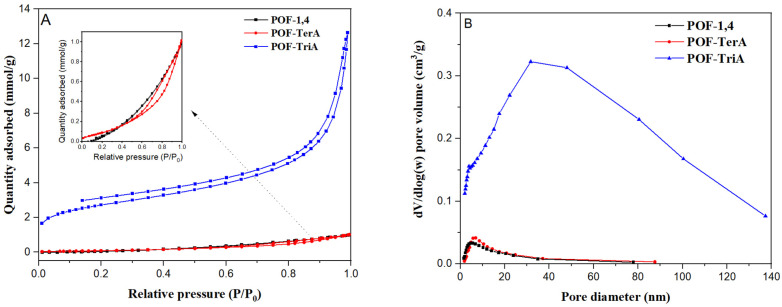
N_2_ isotherm curves (**A**) and BJH pore size distribution (**B**) for the examined samples. The inset of (**A**) presents a zoom of the isotherm curves of the POF-1,4 (black) and the POF-TerA (red).

Pores structures and size significantly affect adsorption performance of such kind of materials [37]. Consequently, the persistent porosity of POFs exposed to environmental pressures was analyzed via N_2_ adsorption–desorption experiments. The textural properties of the samples are reported in Table 2 and Figure 2.

The POF-TriA showed the highest surface area with a type II isotherm and a H4 hysteresis loop typical of macroporous adsorbent with narrow slit-like pores (Figure 2A) [38]. The much higher surface area and pore diameter observed in the POF-TriA could result from the interplay of different factors, structural and synthetic. In particular, the monomer is the most rigid and the one bearing the highest number of cross-linking sites. In addition, its synthesis was carried out for the longest reaction time. The POF-1,4 and the POF-TerA on the contrary, showed a very low surface area with a type III isotherm characteristic of non- or shallowly porous materials for the POF-1,4, whereas the POF-TerA exhibited a type III/IV isotherm with an H3 hysteresis loop associated with the presence of slit-shaped pores (inset of Figure 2A). Accordingly, the pore size distribution of the POF-TriA showed the presence of both meso- and macropores (Figure 2B) with a very larger pore volume compared to the POF-1,4 and the POF-TerA (Table 2). The latter samples possess few pores with a mean pore diameter of 6 nm.

To have further insights into the structural features of the materials obtained, powder X-ray diffraction patterns were acquired (Figure S2). Analysis of the PXRD pattern (Figure S2a) shows that all samples are substantially amorphous, as shown by the absence of Bragg peaks in the 2–90° 2q range. Only POF-TerA showed a weak sharp reflection around 23°, indicating the presence of a very small number of crystalline regions of limited total volume. The above result perfectly agrees with the ones previously reported and highlights that it could be due to the non-uniformity of the structure of this layer of this bidimensional POF [34]. This low crystallinity has been reported for melamine-based POFs [39], although some more crystalline materials have also been reported, with different co-monomer [40,41]. While POF-1,4 did not display any other feature at lower angles, both POF-TerA and POF-TriA showed a strikingly higher scattered intensity below 5°, likely due to the higher surface area. By using the Porod plot (Figure S2b), it was found the scattered intensity of POF-TriA and POF-TerA decay with a q^−3.5^ and q^−4.3^ power law, respectively, suggestive of a fractal-like structure. In the same region, POF-TriA also displayed a peak at 2.36°, corresponding to stacked planes with a periodicity of about 40 Å.

It is worth noting that the amorphous nature of our materials affects directly some properties of the materials connected to their use as sorbent (see later). As reported in the literature, the lack of long-range order in amorphous sorbents, compared with crystalline polymers like COFs, can lead to higher site accessibility and faster diffusion, due to broader pore size distribution. Conversely the well-defined, regular pore arrangement of crystalline COFs, can enhance selectivity and maximum adsorption capacity [31,42].

**Figure 3:**
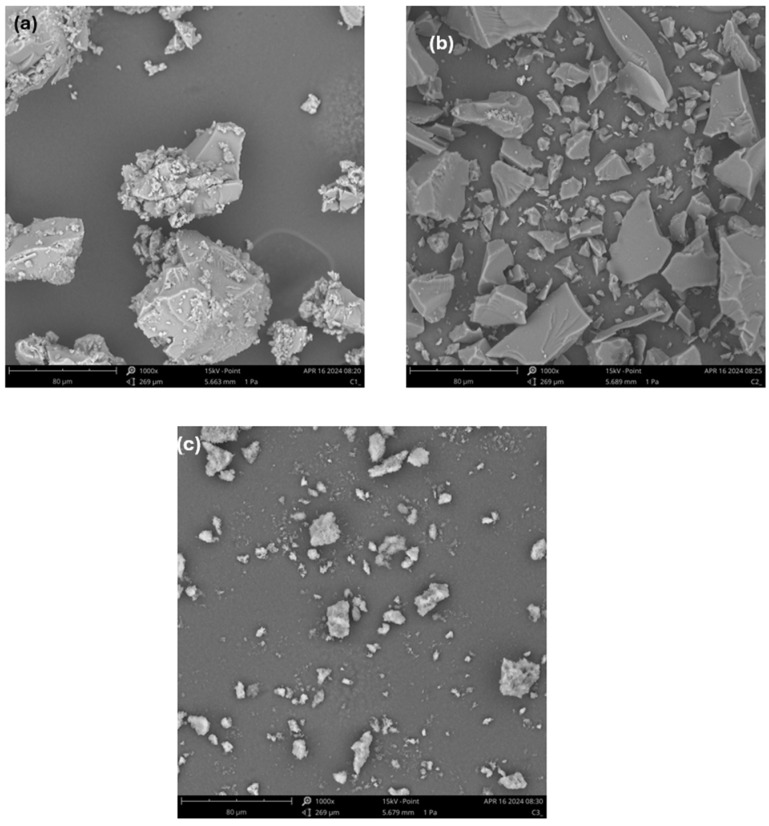
SEM images collected for: (**a**) POF-1,4 (1000×); (**b**) POF-TerA (1000×); (**c**) POF-TriA (1000×).

Morphology of materials was analyzed, performing SEM investigation (Figure 3 and Figure S3). Analysis of SEM micrographs evidences that materials exhibited compact structures, mainly based on grains, in which the visual detection of pores could not be performed.

### 2.2. Dyes Removal in the Presence of POFs: The Effect of Dye Solution Volume

Considering the significantly larger surface area and highly porous structure of POF-TriA, detected by BET analysis, this material was initially used to evaluate its removal efficiency towards different organic dyes. To this aim, increasing volumes of organic dyes solution at 1.8 × 10^−4^ M were contacted with 5 mg of POF-TriA.

**Figure 4:**
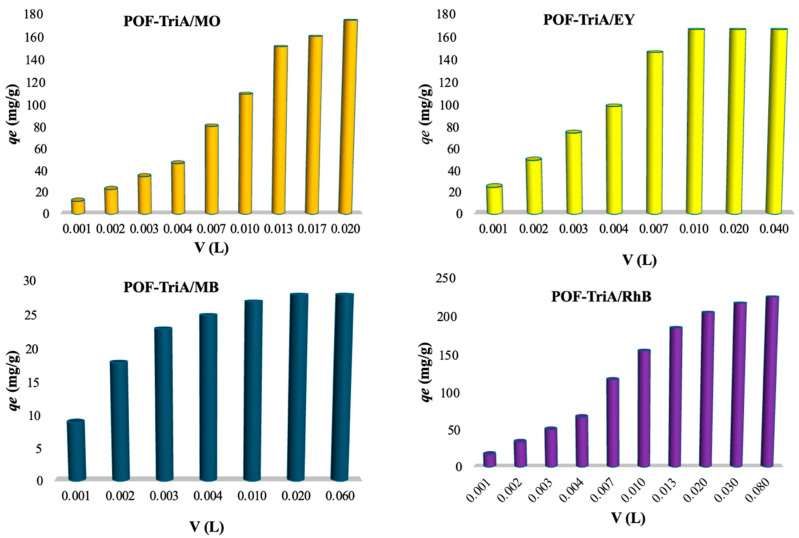
Plots of *q*_e_ (mg/g) as a function of volume (L) of different dyes solutions (1.8 × 10^−4^ M), in the presence of 5 mg of POF-TriA at 298 K. *q_e_* values were reproducible within ±3%.

To determine the residual amount of dye in solution, UV-vis absorption was measured every 24 h, until it stayed constant. This allowed us to calculate the adsorption capacity for all samples at the equilibrium (*q*_e_) by Equation (1), which represents the amount of dye adsorbed per mass of sorbent, expressed in mg/g

(1) $$q_{e}=\frac{\left(C_{0}-C_{e}\right)\times V}{m}$$

where *C*_0_ (mg/L) represents the initial dye concentration, *C_e_* is the residual concentration of dye in solution at the equilibrium (mg/L), *V* is the volume of the solution (L) and *m* is the mass of the adsorbent (g). Results collected, as a function of the different nature of the dye, are reported in Figure 4 and Table S1.

Analysis of data collected evidenced that, in general, *q_e_* increases with the volume of the solution reaching, in most cases a plateau value. The lowest values were collected in the case of MB (*q_e_* at most ~28 mg/g), whereas the highest ones were collected in the presence of RhB (*q_e_* at most ~226 mg/g).

To explain the above data, the structural features of both dyes and POF were considered. We hypothesize that different supramolecular interactions, like hydrogen bond, π-π and van der Waals interactions, as well as ion-π interactions, might operate on the adsorption process. Cationic dyes, namely MB and RhB, probably felt comparable solvation effects, as they had the same charge. However, they differed for the extension of π-surface area, larger in the case of RhB, and for the presence of a hydrogen bond donor group on this latter. Consequently, the higher adsorption capacity determined in the presence of RhB probably derived from a combined action of π-π and hydrogen bond interactions.

These hypotheses are based on structural considerations and are consistent with FTIR spectroscopic evidence (see later); however, computational evidence would be required for a full mechanistic assignment.

For anionic dyes, volume being the same, *q_e_* values resulted higher in the case of MO than in the case of EY. Probably, in the latter case, despite the higher π-surface area, solvation effects, more significant in the case of dianionic EY, prevailed on π-π interactions, determining the trend observed.

**Figure 5:**
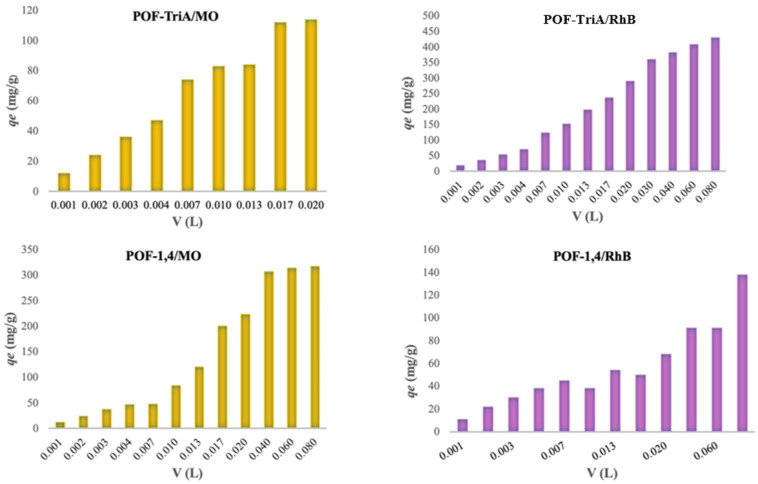
Plots of *q*_e_ (mg/g) as a function of volume (L) of different dyes solutions, in the presence of 5 mg of POF-TerA or POF-1,4. *q_e_* values were reproducible within ±3%.

With the above information in mind, MO and RhB were used as model dyes to evaluate, under the same experimental conditions, the adsorption behavior of POF-1,4 and POF-TerA, changing the volume of the solution. Data collected are reported in Figure 5 and Tables S2 and S3.

Similarly to what was observed in the case of POF-TriA, *q_e_* values increased upon increasing the volume of the solution. Moreover, the sorbents exhibited different behaviors as a function of dyes structure. Indeed, in the case of POF-1,4, *q_e_* ranged from 12 to 317 mg/g for MO and from 11 and 138 mg/g for RhB, revealing a higher efficiency for the anionic dye. Differently, in the case of POF-TerA, the above parameter ranged from 12 and 114 mg/g for MO and from 18 and 429 mg/g for RhB, indicating that this latter POF exhibited a marked affinity for RhB. This hints at an effect deriving from the structure of POF components. Among the POFs tested, POF-1,4 has the lowest aromatic character. Consequently, the high affinity of the above material for anionic dyes could be ascribed to the presence of the aliphatic spacer, that allows reducing the repulsive interactions operating between the anionic dyes and π-electron rich structure of the POF-TriA and POF-TerA. Conversely, the higher efficiency of the latter for cationic dyes can be ascribed to favorable cation π-interactions driving the adsorption process.

Results so far obtained at variable dye solution volume, clearly indicate that, independently from the POF and dye used, good *q_e_* values were collected using 20 mL of dye solution. In the light of this, RhB and MO were considered as model dyes and POF-TriA as model sorbent, to investigate the adsorption process, as a function of the p*H* and ionic strength.

As for the p*H* effect, the adsorption efficiency of model systems was assessed also under basic conditions (p*H* = 10.1). In both cases, increasing the p*H* value, a significant decrease in *q_e_* values was observed (*q*_e_: 128 and 169 mg/g for RhB/POF-TriA and MO/POF-TriA in water, and 80 and 99 mg/g in basic solution), indicating a better efficiency of the tested material in neutral or slightly acidic conditions. Given the nature of the POF functional group, they remain neutral within the p*H* range considered. Consequently, the p*H* dependent change observed could be ascribed to the protonation state of the dye. In particular, in the case of RhB, at acidic p*H* the carboxylate group is partially protonated [43], while at p*H* = 10 it is fully deprotonated, affecting the interaction with the POF surface.

As for the effect of ionic strength, performance of the above model systems was evaluated in NaCl solution at different concentrations, 0.05 M and 0.5 M. In general, *q*_e_ values decreased on going from water to saline solutions (*q*_e_: 128 and 169 mg/g for POF-TriA/RhB and POF-TriA/MO in water; 106 and 71 mg/g in NaCl 0.05 M and 59 and 116 mg/g in NaCl 0.5 M, respectively). To better understand if the influence of ionic strength is due to a electrostatic screening or specific effects of the ions we carried out the same experiments, adsorption of RhB, at the same ionic strength, but changing the salt cation or anion, employing namely KCl 0.5 M and Na_2_SO_4_ 0.17 M The results obtained show similar *q_e_* values, equal to 79 mg/g and 73 mg/g in the presence of KCl and Na_2_SO_4_, respectively. This suggests the occurrence of electrostatic screening effects, ruling out significant specific ion effects.

### 2.3. Dyes Removal: Kinetic Investigation

It is well known that processes aimed at wastewater purification should exhibit fast adsorption. This would entail rapid treatment and the possibility to process large volumes of wastewater. With the above considerations in mind, for all POFs, kinetic investigation as a function of the different nature of dye was performed. In all cases, we investigated adsorption process, using RhB and MO, as model dyes on the grounds of previously discussed results.

Adsorption kinetics were carried out putting in contact 7 mL of a dye solution (1.8 × 10^−4^ M) with 5 mg of POF, at 298 K. *q_e_* values were obtained time by time and trends of *q_e_* as a function of time were analyzed using both pseudo-first and pseudo-second order kinetic models (see Supplementary Information for details).

Figure S4 displays kinetic trends, whereas fitting parameters, namely *q*_e_, *k*_1_ (pseudo-first order kinetic constant) and *k*_2_ (pseudo-second order kinetic constant) obtained as a function of different nature of POF, dye and kinetic model are reported in Table 3.

In general, higher correlation coefficients (R^2^) were obtained using the pseudo-second order model. However, high R^2^ values collected also using the pseudo-first order model, suggest the involvement of different mechanisms in which the adsorption through pores can also play a role [44], entailing a complex adsorption mechanism potentially involving both surface binding and pore diffusion, rather than a single, rate-limiting step. To verify the above hypothesis, data collected were further analyzed using the intra-particle diffusion model [45,46]. This model foresees that adsorption process might feature by three steps: (i) the external surface diffusion rate, where adsorbate molecules interact with active sites on the adsorbent (high slope); (ii) the second and third steps that consist of diffusion in the macro- and micropores of the material. In general, rates decrease going from the first to the third step, because of restrictions deriving from pores size of the adsorbent, as well as of the occurrence of repulsive interactions, occurring during the diffusion. Analysis of collected data (Figure S5 and Table S4), on the grounds of the above model, indicates that in major cases, the intra-particles diffusion fitting lines did not cross the origin, supporting the hypothesis that adsorption is driven by at multiple steps, like surface adsorption and pore diffusion. Analysis of *k*_2ip_ values reported in Table S4 evidences that the rate of the diffusion step can be related to the nature of the adsorbent as it changes according to the following trend: POF-TerA < POF-1,4 < POF-TriA. The above trend perfectly recalls the one of porosity of the adsorbents (see Table 2). On the other hand, adsorbent being the same (e.g., POF-TerA or POF-TriA), *k*_2ip_ significantly increases going from RhB to MO, indicating the occurrence of a faster diffusion process in the case of the smaller adsorbate (d = 16 and 12.35 Å for RhB [47] and MO [48], respectively).

Comparing POFs performance evidences a faster adsorption process in the presence of POF-TriA (see *k*_2_ values in Table 3), that also worked better in the presence of MO than RhB. Dyes being the same, MO, *k*_2_ values changed along the following trend: POF-TriA > POF-TerA > POF-1,4. As for RhB, kinetic investigation was not performed in the presence of POF-1,4, because of the low adsorption efficiency measured in the preliminary tests.

The above trends can be related to POFs porosity. Indeed, independently of the nature of the organic dyes, adsorption occurs faster in the presence of POF-TriA, that also exhibits the highest porosity. On the other hand, in the case of POF-1,4 and POF-TerA, comparable *k_2_* values were collected, according to BET surface area (see Table 2). On the other hand, transitioning from POF-1,4 to POFs with a more extended aromatic structure leads to an increase in adsorption rate.

Kinetic investigation was also performed using dye mixtures that better represent a real situation. To this aim, POF-TerA and POF-TriA were used as adsorbents, in the presence of RhB and MO mixtures (1.8 × 10^−4^ M). Before the measurements, we verified the absence of superposition between the spectra of the two dyes, as shown in Figure S6. Therefore, we determined the residual amount of each dye in solution by UV-vis spectroscopy. Fitting parameters calculated by using both first and second order kinetic models are reported in Table 4, whereas kinetic plots are displayed in Figure S7.

Analysis of correlation coefficients allows stating that also in the presence of mixtures of dyes, the pseudo-second order kinetic model better described experimental trends. However, once again, data collected for POF-TerA/MO and POF-TerA/RhB show high R^2^ values also using pseudo-first order model, accounting for the occurrence of different mechanisms. Independently from the nature of dye, kinetic constants detected in the presence of POF-TriA were higher than the ones collected in the presence of POF-TerA. Furthermore, regardless of the nature of material, adsorption process was faster in the case of MO than in the case of RhB.

Comparison with *k*_2_ values collected in the case of single dye solutions demonstrates that POF-TerA exhibited comparable performance (*cfr*, Table 3 and Table 4), allowing to exclude the presence of competitive processes. Interestingly, in the case of POF-TriA, for both dyes, a significant increase in the adsorption rate was detected in the case of the mixtures, evidencing the occurrence of a positive cooperative effect.

### 2.4. Dyes Removal in the Presence of POFs: The Study of the Adsorption Isotherms

To gain insights on the properties of the adsorbent materials and the mechanism of adsorption, the adsorption isotherms were determined. It is well known that this kind of analysis allows identifying the relationship between the adsorption capacity (*q_e_*) at the equilibrium, at constant temperature, and the equilibrium solute concentration in solution (*C_e_*), allowing to evaluate the affinity between the adsorbent and the solute. Different models have been developed, and in this case, we firstly considered POF-TriA, in the presence of all dyes, analyzing the results with different adsorption isotherms, among which the Langmuir [49] and Freundlich [50] ones gave rise to the high correlation coefficients and will be further discussed (see Supplementary Information for details).

Results collected according to the Langmuir model, are reported in Table 5, whereas the ones obtained using the Freundlich model are displayed in Table S5. Plots corresponding to the binding isotherms are reported in Figure S8.

In Table 5, *q_m_* represents the maximum adsorption capacity, whereas *K_L_* is the Langmuir constant that accounts for the affinity between the solute and the active sites on the adsorbent.

Comparison among data collected evidences how in all cases, dyes adsorption was better described by the Langmuir than Freundlich model, as accounted for by the higher correlation coefficients. This result indicates that the surface of the adsorbent was homogeneous containing only one type of binding sites and the adsorption energy is constant.

*q*_m_ values range from 36 mg/g for POF-TriA/MB up to 472 mg/g for POF-TerA/RhB. Data collected evidence the high efficiency of aromatic POFs, i.e., POF-TriA and POF-TerA, for RhB (*q*_m_: 233 and 472 mg/g in the presence of POF-TriA and POF-TerA, respectively). This result perfectly recalls the one collected performing investigation at different volumes of dye solution and supports the hypothesis that adsorption process was mainly driven by cation-π interactions. The significant decrease in *q_m_* values, going from POF-TriA/RhB to POF-TriA/MB, can be ascribed to the decrease in conjugate system of MB (compared to RhB), that gives rise to the occurrence of feebler π-π interactions. A similar behavior has been recently detected studying adsorption performance of 3D-COOH-COF towards the same organic dyes and represents the relevance of π-π interactions in driving the adsorption process [48,51].

On the other hand, for the above materials, *q*_m_ decreased on going from RhB to anionic dyes and, in the case of POF-TriA, it stayed constant independently from the nature of the anionic dye structure (MO or EY).

Anionic dye being the same (MO), analysis of *q*_m_ values as a function of POF nature sheds light on the role played by aromaticity and electron density of the POF. Indeed, a significantly high *q*_m_ value (329 mg/g) was detected in the presence of POF-1,4. However, the above parameter significantly decreases in the presence of POF-TriA and POF-TerA (*q*_m_: 175 and 88 mg/g for POF-TriA and POF-TerA, respectively). According to the previous hypothesis, this trend reflects a gradual increase in the repulsive interaction between electron rich species (POF surface and aromatic anionic dyes), counterbalancing the positive effect of π-π interactions. A schematic summary of the relationship between aromatic/aliphatic nature of the POF spacer and adsorption performance is reported in Scheme S4.

### 2.5. Dyes Removal in the Presence of POFs: The Study of the Temperature Effect

Adsorption process was also investigated as a function of temperature. To this aim, solutions of RhB and MO were equilibrated with all POFs, under the same experimental conditions previously described, for 24 h at different temperatures in the range 293–323 K (Figures S9 and S10). Then, thermodynamic parameters associated with the adsorption process were calculated [52,53].

To determine the thermodynamic parameters, the distribution coefficient *K*_d_ = (*q_e_*/C_e_) was used as approximate equilibrium parameter, assuming quasi-linear behavior under the conditions used in these experiments. *K*_d_ was first converted into the dimensionless constant [54]. *K*^0^ as reported in the calculation details in Supplementary Information. Given the approximations used, the thermodynamic parameters Δ*H*, Δ*S* and Δ*G* should be considered apparent values, valid only on the conditions employed in this study.

Values collected, together with ΔG values at 298 K, calculated according to the van’t Hoff equation are reported in Table 6.

In the case of POF-1,4/MO, *q*_e_ values were not significantly affected by temperature and, consequently thermodynamic parameters were not calculated. As for the other systems, analysis of collected results evidences how independently from the dye and POF nature, *q*_e_ gradual increased from 273 up to 323 K, indicating the endothermic nature of the adsorption process. In all the other cases, the magnitude of the adsorption enthalpies was consistent with a physisorption process. On the other hand, analysis of Δ*G* values (Table 6) demonstrates that adsorption process was always spontaneous and allows identifying POF-TriA as the most selective adsorbent material, as accounted for by the significant difference detected in Δ*G* values between the cationic and anionic dye.

To further investigate the mechanism of adsorption, and the interactions between sorbent and adsorbate driving the process, we obtained the FTIR spectra of the materials after adsorption of selected dyes and compared them with the ones relevant to the pristine POFs and dye. We recorded the spectra of POF-TriA after adsorption of RhB and MO, as well as POF-TerA with RhB and POF 1,4 with MO. The stacked spectra are reported in Figure S11. Examination of the spectra of POF-TriA upon adsorption of RhB (Figure S11a), shows that the band relevant to the O-H stretching of RhB shifts from 3434 cm^−1^ to lower frequencies at 3408 cm^−1^. Similarly, the C=O stretching band of the POF, upon adsorption shifts from 1590 to 1580 cm^−1^, and both observations suggest the occurrence of hydrogen bonding between POF-TriA and RhB. Similar considerations can be made when the same material adsorbs an anionic dye like MO, in which case, as shown in Figure S11b, the C=O stretching band moves from 1590 cm^−1^ to 1580 cm^−1^. On the other hand, looking at the spectra reported in Figure S11c reveals that upon adsorption of RhB by POF TerA, the O-H stretching band of the dye, once again moves at lower frequencies, from 3434 cm^−1^ to 3393 cm^−1^. In addition, the C=N stretching band of the POF shifts from 1595 cm^−1^ to 1575 cm^−1^; according to reports in the literature [28,55], such change can be attributed to the establishment of π-π interaction involving the POF and the dye. Finally, considering the variation in IR spectra when POF-1,4 adsorbs MO, Figure S11d, reveals that the N-H stretching band of the POF moves from 3430 cm^−1^ to 3416 cm^−1^, as a consequence of hydrogen bonding, and the C=C stretching bands of both the POF and the dye shift from 1643 cm^−1^ and 1605 cm^−1^ respectively, to 1596 cm^−1^, clearly suggesting the occurrence of π-π interactions.

**Scheme 2:**
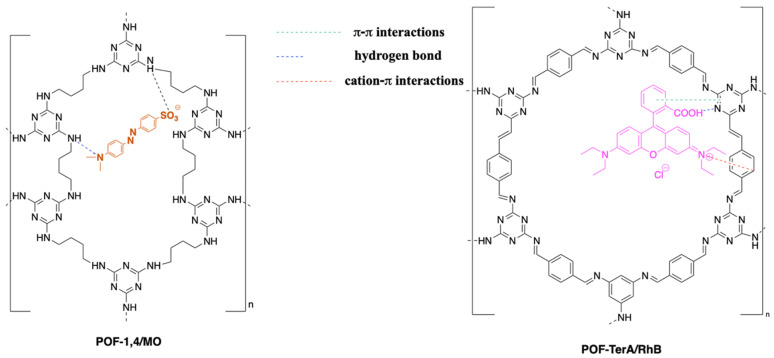
Representation of POF-dye interaction in POF-1,4/MO and POF-TerA/RhB systems.

Based on all the above considerations, it is possible to argue that main interactions working on the adsorption of the studied dyes were hydrogen bond, π-π and cation-π interactions. Similar findings have been reported for the adsorption of dyes by different COFs and dyes [56,57]. The relevance of each of these interactions probably changes on the grounds of POF and dye structure. In the light of the above premises, the high affinity of POF-1,4 for MO was probably ascribable to the establishment of hydrogen bond interactions. In this case, the aliphatic nature of the co-monomer significantly decreases the possibility of electronic repulsion between the overall negatively charged dye and the high π-electron density characterizing POF-TerA and POF-TriA. On the other hand, in the case of RhB and POF-TerA or POF-TriA, together with hydrogen bond, also π-π and cation-π interactions played a pivotal role (Scheme 2).

### 2.6. POF Recycling

In the light of all collected results, indicating POF-TriA as the best performing porous material, the possibility of its recycling was evaluated in the adsorption of RhB and MO. To this aim, to set the best conditions, different protocols were used. In the first attempt, taking into consideration the results collected by us, using supramolecular ionogels for the removal of organic contaminants from wastewater [6,58], the adsorbent after the first cycle was put in contact with a fresh solution of dye, without intermediate washing. However, using these conditions, no significant adsorption occurred. Then, in a second attempt, POF was filtered and washed by centrifugation with portions of 1 mL of methanol (2 times), until the absorbance of the methanol phase was negligible. The material was dried in an oven, at 70 °C, overnight and reused for further adsorption.

To verify that the sorbent kept its integrity after the washing step, we recorded the FTIR spectrum and compared it with the one of the pristine materials, showing that they were fully superimposable (Figure S12).

**Figure 6:**
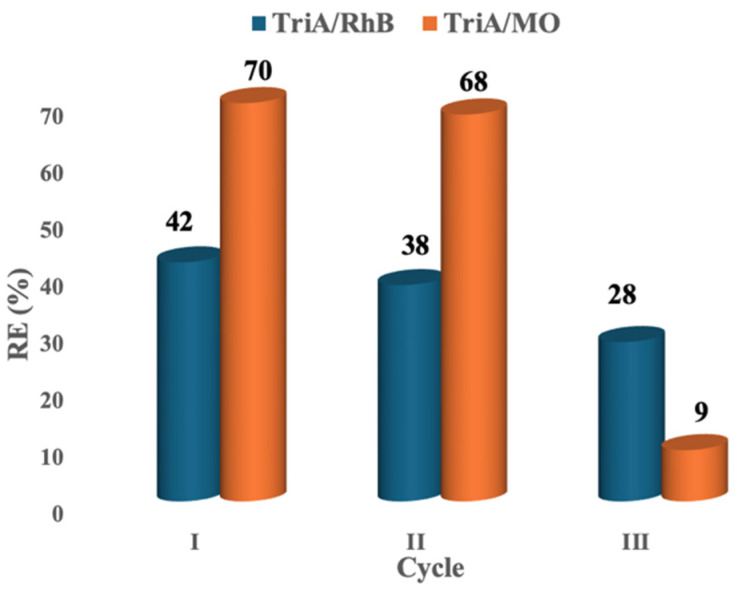
Removal efficiency (RE%) determined at 24 h, for POF-TriA/RhB and POF-TriA/MO (1.8 × 10^−4^ M; 20 mL; 5 mg) after different cycle of reusing.

In Figure 6, the collected removal efficiency (RE, %) after each cycle, are reported.

In both cases, performance of POF-TriA stayed constant for the first two cycles. In third cycle, a decrease in AE values was detected that proved more significant in the case of MO than RhB. However, it is worth mentioning that because of the physical adsorption of dyes on the tested material and, differently from numerous reports in the literature, reuse of our material can be performed, employing a solvent admitted by the solvent selection guides [59], and avoiding the use of more impacting solvent systems, like acidic or basic solutions.

**Figure 7:**
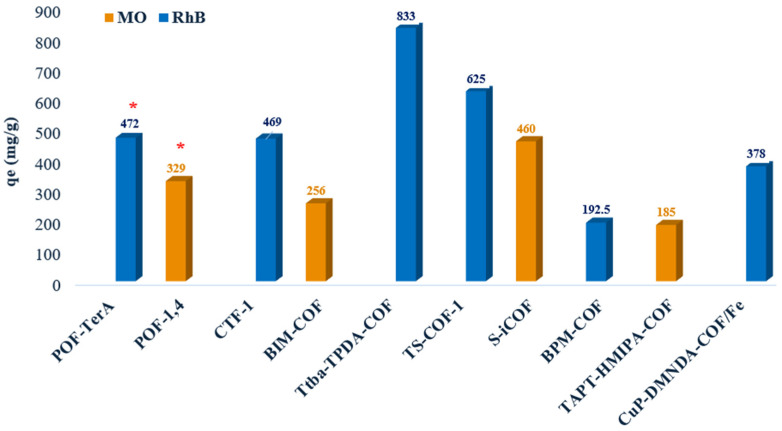
Comparison among POF-1,4/MO and POF-Tera/RhB with data previously reported in the literature (* = data collected in this work).

To have a better evaluation of our systems, we compared our best results obtained for POF-1,4/MO and POF-TerA/RhB, with the ones previously reported in the literature (Figure 7 and Table S6). It is important to note that these results were not obtained under the same experimental conditions, such as initial dye concentration or sorbent dosage. Nevertheless, a comparison of the performance could be still useful.

Analysis of data displayed in Figure 7 evidence how performance of POF-1,4/MO was, in most cases, comparable or higher with respect to data reported in the literature. In particular, the performance of POF-1,4 was significantly superior to the one of the benzimidazole-based COF, BIM-COF reported by Xu [60]. It is important to note that these results are reached under different p*H* conditions, as the latter material achieves 256 mg/g of adsorption capacity in acidic solutions. Similar consideration can be made by comparing POF-1,4 with the triarylamine-based COF TAPT-HMPA-COF [61]. The only exception was represented by the cationic S-iCOF, that exhibits a capacity equal to 460 mg/g and superior selectivity for anionic dyes, due to charge complementarity [62], although in this latter case, reusing the spent adsorbent requires a multiple sequential treatment with aqueous NaNO_3_, ethanol and finally HCl 5 M. As for RhB, performance of our system, POF-TerA/RhB, was comparable to the bidimensional triazine-based CTF-1 [63] and CuP-DMNDA-COF/Fe, an imine linked porphyrin COF doped with Fe(II) [64], although in this latter case higher recyclability was reported. On the other hand, the performance of POF-TerA was lower than the one exhibited by the two-dimensional triazine based COF, Ttba-TPDA-COF [65] and the bidimensional TS-COF-1 [66]. Similar considerations can be drawn from considering the performance of our POF-Tria, in which case, the longer adsorption time is compensated by simpler washing, requiring no acid treatment. Finally, reusing the sorbent is well in line with the other materials reported in Table S6.

## 3. Materials and Methods

### 3.1. Materials

Melamine (99%), trimesic acid (95%), terephthalaldehyde (99%), 1,4-dibromobutane (99%), methyl orange, rhodamine B, methylene blue, eosin yellow, DMSO, acetone and methanol were obtained from commercial sources and used without further purification.

POF-1,4 [33], POF-TerA [34] and POF-TriA [35] were prepared following previously reported procedures. Further details are reported in Supplementary Information.

### 3.2. Characterization

The Brunauer–Emmett–Teller (BET) surface area and the Barrett–Joyner–Halenda (BJH) pore size distribution (using the desorption curve) of the samples were measured by physisorption of N_2_ at −196 °C using a Micromeritics Tristar II Plus 3020 instrument, (Norcross, GA, USA) outgassing the samples at 60 °C overnight.

X-ray diffraction (XRD) was acquired on powder samples using a Rigaku MiniFlex 600 diffractometer (Akishima-shi, Japan) with Ni-filtered Cu K_α_ radiation.

SEM images were obtained on a PRO X PHENOM electronic scanning microscope, operating at 10 kV (Eindhoven, The Netherlands).

FT-IR spectra were obtained from KBr pellets.

### 3.3. Adsorption Tests

Aqueous solutions of dyes were prepared by dilution of suitable stock solutions. The initial concentration of the aqueous solutions of dyes was equal to 1.8 × 10^−4^ M. Typical adsorption tests were carried out in vials by placing the suitable amount of POF in contact with a solution of dye. All experiments were conducted under static conditions in unbuffered solutions. The initial p*H* was approximately 6.7 and no significant variations were detected after the adsorption test.

After a suitable time, an aliquot of solution was withdrawn, diluted and then the relevant UV-spectrum was recorded. The residual concentrations of dyes were determined based on previously determined calibration curves.

Recycling experiments were carried out by filtering off the solid, after an adsorption run, and suspending it in 1 mL of methanol. Centrifugation at 5000 rpm (5 min) and removal of the supernatant yielded the solid which was put in contact with a fresh batch of dye solution. The residual concentrations of dyes were determined as described above and the procedure was repeated (generally no more than 3 times) until the absorbance of the methanol phase was negligible. No significant mass loss was detected at each reuse cycle.

Adsorption tests at variable temperature were carried out in a thermostatic bath able to keep temperature constant within ±0.1 °C.

### 3.4. Adsorption Isotherms

The experiments to determine the adsorption isotherms were carried out at 25 °C, for a contact time of at least 24 h, following the same procedure described above for the adsorption tests. In each experiment, the contact time was chosen, by preliminary kinetic tests, ensuring that equilibrium was reached, and that the residual concentration of dye did not significantly vary at longer times. Different volumes and initial concentration of the dye solution were used. Experiments for adsorption isotherms were conducted in duplicate. The results of these experiments were fitted according to Langmuir and Freundlich models [67].

### 3.5. Kinetic Adsorption Tests

Kinetic adsorption tests were carried out by independent adsorption runs at variable contact time between POFs and dye solutions. The mass of POF used was 5 mg and the volume of dye solution was 7 mL (1.8 × 10^−4^ M). The residual concentration of dye in solution was evaluated as described above.

## 4. Conclusions

In summary, in this work, we successfully analyzed the effect of structural features of three melamine-based POFs on the adsorption performance towards cationic and anionic organic dyes. These materials were prepared using aliphatic (1,4-dibromobutane) and aromatic (terephthaladehyde and trimesic acid) co-monomers.

Data collected show that the propensity of POFs for cationic or anionic dyes depended on the aromaticity degree of the adsorbent, with POF-1,4 showing the better efficiency in removing the anionic dye MO. Increasing the aromaticity degree of the POF leads to higher efficiency in the removal of cation dyes, and to a parallel increase in adsorption rate.

In all cases, the adsorption process was endothermic in nature, with POF-TriA showing the highest selectivity, as accounted for by significant differences in Δ*G* values corresponding to MO and RhB.

This material was used with mixtures of dyes without loss in performance, and it enhanced adsorption rates, which suggest potential application of this material in the treatment of real wastewaters. FT-IR analysis of the adsorbent, before and after the adsorption process, supports the hypothesis that interaction with dyes is driven by hydrogen bond, π-π and cation-π interactions. The occurrence of the above non-covalent interactions allowed to reuse the POF-TriA at least three times, after regeneration with a small amount of methanol, and this represents a significant difference with respect to regeneration methodologies frequently reported in the literature, requiring harsher acidic or basic treatments, which constitutes a commonly encountered limitation. This suggests also potential benefits for possible scale-up.

Overall, collected data in this work offer the opportunity to systematically relate the adsorption efficiency to structural features of melamine-based POFs. This kind of investigation can represent a useful support to design POF-based adsorbent materials for specific class of contaminants, avoiding time consuming and environmentally impacting synthesis and investigations.

## Figures and Tables

**Table 1:** Decomposition temperatures (*T_d_*) obtained from DTGA traces for different POFs (weight loss % are reported in brackets).

	*T_d_*_1_ (°C)	*T_d_*_2_ (°C)	*T_d_*_3_ (°C)
POF-1,4	250 (21%)	304 (32%)	484 (66%)
POF-TerA	127 (15%)	378 (34%)	549 (57%)
POF-TriA	360 (31%)	442 (65%)	533 (88%)

**Table 2:** Textural properties of the examined POFs.

	BET Surface Area (m^2^/g)	Mean Pore Diameter (nm)	Pore Volume (cm^3^/g)
**POF-1,4**	7 ± 2	6.0 ± 0.2	0.03 ± 0.02
**POF-TerA**	9 ± 2	6.1 ± 0.2	0.03 ± 0.02
**POF-TriA**	425 ± 2	37.0 ± 0.2	0.39 ± 0.02

**Table 3:** Fitting parameters for the adsorption kinetics, calculated according to pseudo-first and pseudo-second order kinetic model, as a function of POF and dye nature. Measurements performed at 298 K, contacting 7 mL of a dye solution (1.8 × 10^−4^ M) with 5 mg of POF.

	Pseudo-First Order			Pseudo-Second Order		
Dye	*k*_1_ (min^−1^)	*q_e_* (mg/g)	R^2^	*k*_2_ (g·mg^−1^·min^−1^)	*q_e_* (mg/g)	R^2^
**POF-1,4**						
MO	(1.1 ± 0.06) × 10^−2^	63 ± 1	0.966	(1.6 ± 0.2) × 10^−4^	77 ± 1	0.988
**POF-TerA**						
MO	(15 ± 1) × 10^−3^	42 ± 1	0.929	(3.6 ± 0.3) × 10^−4^	50 ± 1	0.977
RhB	(8.9 ± 0.5) × 10^−3^	48 ± 1	0.974	(1.3 ± 0.1) × 10^−4^	63 ± 1	0.982
**POF-TriA**						
MO	(56 ± 5) × 10^−3^	75 ± 1	0.870	(1.04 ± 0.06) × 10^−3^	82 ± 1	0.976
RhB	(40 ± 5) × 10^−3^	87 ± 2	0.916	(6.0 ± 0.7) × 10^−4^	97 ± 2	0.973

**Table 4:** Fitting parameters for the adsorption kinetics, as a function of POF and dye nature, according to pseudo-first and pseudo-second order kinetic model, performed in the presence of dye mixtures (1.8 × 10^−4^ M), at 298 K.

	Pseudo-First Order			Pseudo-Second Order		
Dye	*k*_1_ (min^−1^)	*q_e_* (mg/g)	R^2^	*k*_2_ (g·mg^−1^·min^−1^)	*q_e_* (mg/g)	R^2^
**POF-TerA**						
MO	(1.9 ± 0.2) × 10^−2^	60 ± 1	0.909	(3.5 ± 0.4) × 10^−4^	69 ± 1	0.967
RhB	(9.5 ± 0.6) × 10^−3^	56 ± 1	0.974	(1.2 ± 0.1) × 10^−4^	73 ± 1	0.987
**POF-TriA**						
MO	(1.2 ± 0.1) × 10^−2^	77 ± 1	0.818	(2.8 ± 0.1) × 10^−3^	81 ± 1	0.979
RhB	(59 ± 7) × 10^−3^	96 ± 2	0.811	(8.0 ± 1) × 10^−4^	105 ± 1	0.945

**Table 5:** Fitting parameters for the Langmuir model, as a function of POF and dye nature.

	*K_L_* (L/mg)	*q*_m_ (mg/g)	R^2^
**POF-1,4**			
MO	0.6 ± 0.1	329 ± 12	0.978
**POF-TerA**			
RhB	0.13 ± 0.02	472 ± 16	0.991
MO	0.66 ± 0.08	88 ± 2	0.986
**POF-TriA**			
RhB	0.24 ± 0.04	233 ± 9	0.980
MB	0.07 ± 0.02	36 ± 3	0.908
MO	1.8 ± 0.2	175 ± 2	0.967
EY	0.4 ± 0.1	170 ± 13	0.865

**Table 6:** Thermodynamic parameters for RhB and MO adsorption process as a function of different POF nature.

Dye	Δ*H* (kJ/mol)	Δ*S* (J/K mol)	Δ*G*_298K_ (kJ/mol)
**POF-TerA**			
RhB	60 ± 10	280 ± 20	−17
**POF-TriA**			
RhB	21 ± 4	130 ± 14	−18
MO	27 ± 5	160 ± 20	−21

## Data Availability

Data are available in Supplementary Material.

## Supplementary Materials

The following supporting information can be downloaded at: https://www.mdpi.com/article/10.3390/molecules31122022/s1. POFs preparation, Kinetic models, Binding Isotherm models, Intra-particle diffusion model. Determination of thermodynamic parameters. Table S1: *q_e_* values determined in the presence of POF-TriA, as a function of volume of solutions of different dyes. Table S2: *q_e_* values determined in the presence of POF-TerA, as a function of volume of solutions of different dyes. Table S3: *q_e_* values determined in the presence of POF-1,4, as a function of volume of solutions of different dyes. Table S4: Intra-particle diffusion in adsorption all dyes as a function of different POFs. Table S5: Fitting parameters for the Freundlich model, as a function of POF and dye nature. Table S6: Comparison among data collected using POF-1,4/MO and POF-Tera/RhB and data previously reported in literature. Figure S1: TGA and DTGA traces for (a) POF-1,4; (b) POF-TerA; (c) POF-TriA. Figure S2: (a) Measured PXRD patterns of POFs; (b) Porod plot of POF-TFA and POF-TerA, with power-law regressions shown in black. Both axes are in logarithmic scale. Figure S3: SEM images collected for: (a) POF-1,4 (5000×); (b) POF-TerA (5000×); (c) POF-TriA (5000×). Figure S4: Plots of q_t_ values as a function of the time, at 298 K, for all POFs, in the presence of RhB or MO. Figure S5: Intraparticle diffusion models for all POFs in the presence of MO or RhB at 1.8 × 10^−4^ M. Figure S6: Superimposed adsorption spectra of MO and RhB. Figure S7: Plots of qt values as a function of the time, at 298 K, for POF-TerA and POF-TriA, in the presence of mixtures of RhB or MO. Figure S8: Plots of *q_e_* values as a function of C_e_, at 298 K, for all POFs. Figure S9: Plots of *q_e_* values as a function of the temperatures for all POFs (5 mg), in the presence of RhB or MO solution (1.8 × 10^−4^ M; 20 mL). Figure S10: Plots of ln (*q_e_*/C_e_) as a function of 1/T for all POFs (5 mg), in the presence of RhB or MO solution (1.8 × 10^−4^ M; 20 mL). Figure S11: Stacked IR spectra of POFs upon adsorption of dyes and pristine materials. Figure S12: Stacked spectra of pristine and recycled POF-TriA. References [33,34,35,45,49,50,56,60,61,62,63,64,66,67] are cited in the Supplementary Materials.
