# Supplementary material for: Melamine-Based Porous Organic Frameworks as Adsorbent Materials for the Removal of Organic Dyes from Wastewater
Source: Molecules. 2026 Jun 9;31(12):2022. doi: 10.3390/molecules31122022 (PMC13304876; doi:10.3390/molecules31122022)
Supplement: Supplementary file 1 [file molecules-31-02022-s001.zip › molecules-4267137-supplementary.pdf]

Supplementary Material

Melamine-Based Porous Organic Frameworks as Adsorbent Materials for the Removal of Organic Dyes from Wastewater

Salvatore Marullo [1,*], Giovanna Raia [1], Roberto Fiorenza [2], Martina Maria Calvino [3], Francesco Giannici [3], Giuliana Impellizzeri [2] and Francesca D'Anna [1,*]

[1] Dipartimento STEBICEF, Università degli Studi di Palermo, Viale delle Scienze Ed. 17, 90128 Palermo, Italy
[2] Dipartimento di Scienze Chimiche, Università degli Studi di Catania, Via A. Doria 6, 95125 Catania, Italy
[3] Dipartimento DiFC, Università degli Studi di Palermo, Viale delle Scienze Ed. 17, 90128 Palermo, Italy
[4] CNR-IMM, Via Santa Sofia 64, 95123 Catania, Italy
* Correspondence: salvatore.marullo@unipa.it (S.M.); francesca.danna@unipa.it (F.D.)

POFs preparation	Psge S2
Kinetic models	Psge S3
Binding Isotherm models	Psge S3
Intra-particle diffusion model	Psge S4
Determination of thermodynamic parameters	Psge S6
**Table S1.** *qe* values determined in the presence of POF-TriA, as a function of volume of solutions of different dyes.	Psge S7
**Table S2.** *qe* values determined in the presence of POF-TerA, as a function of volume of solutions of different dyes.	Psge S8
**Table S3.** *qe* values determined in the presence of POF-1,4, as a function of volume of solutions of different dyes.	Psge S9
**Table S4.** Intra-particle diffusion in adsorption all dyes as a function of different POFs.	Psge S10
**Table S5.** Fitting parameters for the Freundlich model, as a function of POF and dye nature.	Psge S10
**Table S6.** Comparison among data collected using POF-1,4/MO and POF-Tera/RhB and data previously reported in literature.	Psge S11
**Figure S1.** TGA and DTGA traces for: **a)** POF-1,4; **b)** POF-TerA; **c)** POF-TriA.	Psge S12
**Figure S2.** *a*) Measured PXRD patterns of POFs; *b*) Porod plot of POF-TFA and POF-TerA, with power-law regressions shown in black. Both axes are in logarithmic scale.	Psge S12
**Figure S3.** SEM images collected for: **a)** POF-1,4 (5000x); **b)** POF-TerA (5000X); **c)** POF-TriA (5000X).	Psge S13
**Figure S4.** Plots of q_t values as a function of the time, at 298 K, for all POFs, in the presence of RhB or MO.	Psge S14
**Figure S5.** Intraparticle diffusion models for all POFs in the presence of MO or RhB at 1.8• 10^{-4} M.	Psge S15
**Figure S6**. Superimposed adsorption spectra of MO and RhB.	Psge S15
**Figure S7.** Plots of q_t values as a function of the time, at 298 K, for POF-TerA and POF-TriA, in the presence of mixtures of RhB or MO.	Psge S15
**Figure S8.** Plots of *qe* values as a function of C_e, at 298 K, for all POFs. q_e values were reproducible within ± 3%.	Psge S16
**Figure S9.** Plots of q_e values as a function of the temperatures for all POFs (5 mg), in the presence of RhB or MO solution (1.8 · 10^{-4} M; 20 mL). q_e values were reproducible within ± 3%.	Psge S16

Figure S10. Plots of ln (q_e/C_e) as a function of 1/T for all POFs (5 mg), in the presence of RhB or MO solution (1.8 · 10^{-4} M; 20 mL). Psge S17
Figure S11. Stacked IR spectra of POFs upon adsorption of dyes and pristine materials Psge S18
Figure S12. Stacked spectra of pristine and recycled POF-TriA. Psge S18

POFs preparation

POFs were synthesized using previously reported procedures. In details, POF-1,4 was obtained reacting melamine, in DMSO solution, in the presence of 1,4-dibromobutane (Scheme S1).[1]

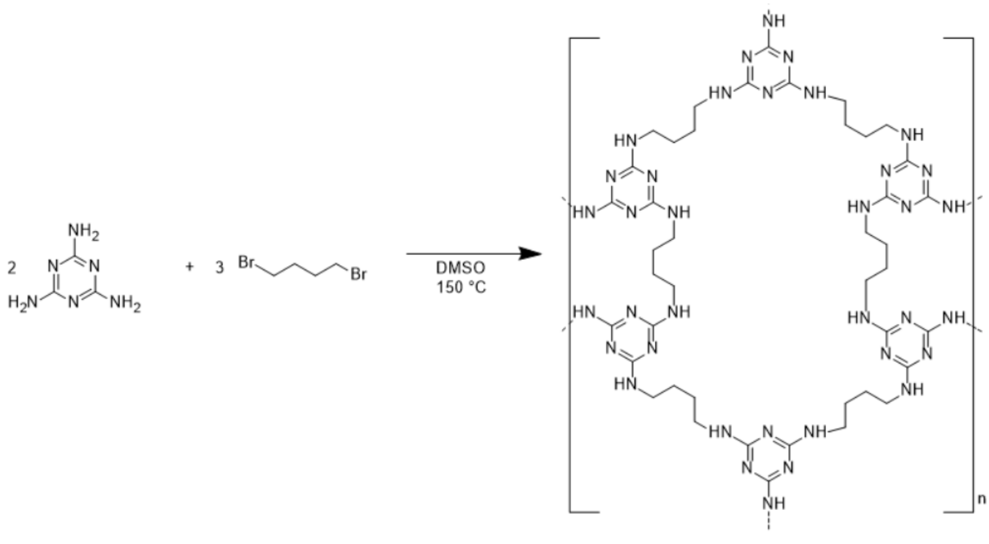

POF-1,4

Scheme S1. Synthesis of POF-1,4.

POF-TerA was obtained by the reaction between melamine and terephthalaldehyde (Scheme S2).[2]

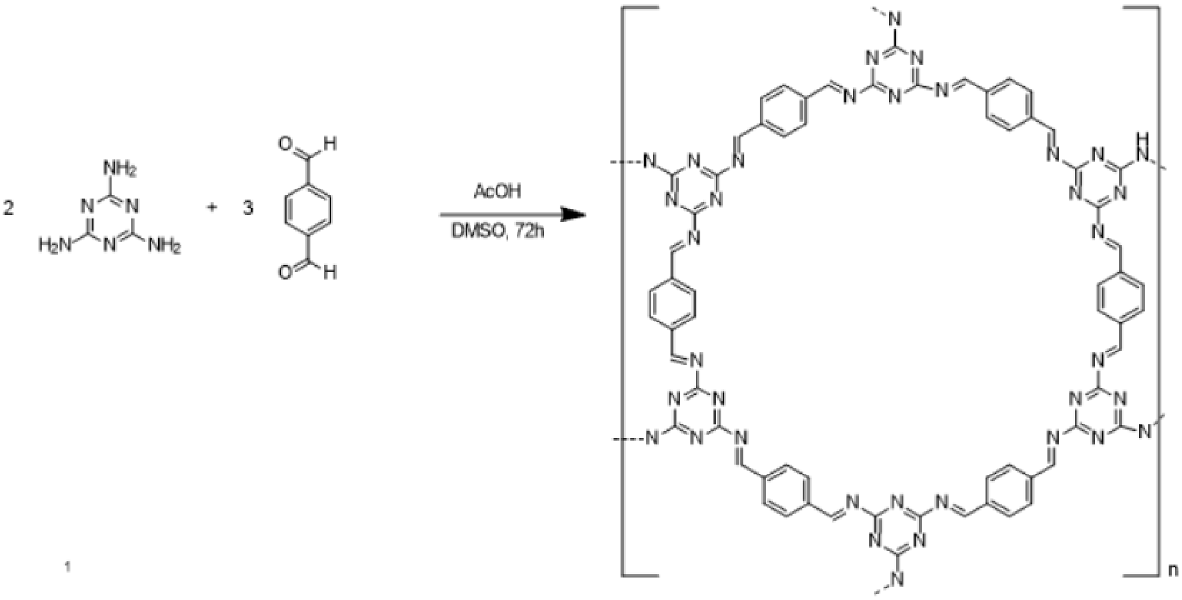

POF-TerA

Scheme S2. Synthesis of POF-TerA.

Finally, POF-TriA was obtained from the reaction between melamine and trimesic acid. (Scheme S3).[3]

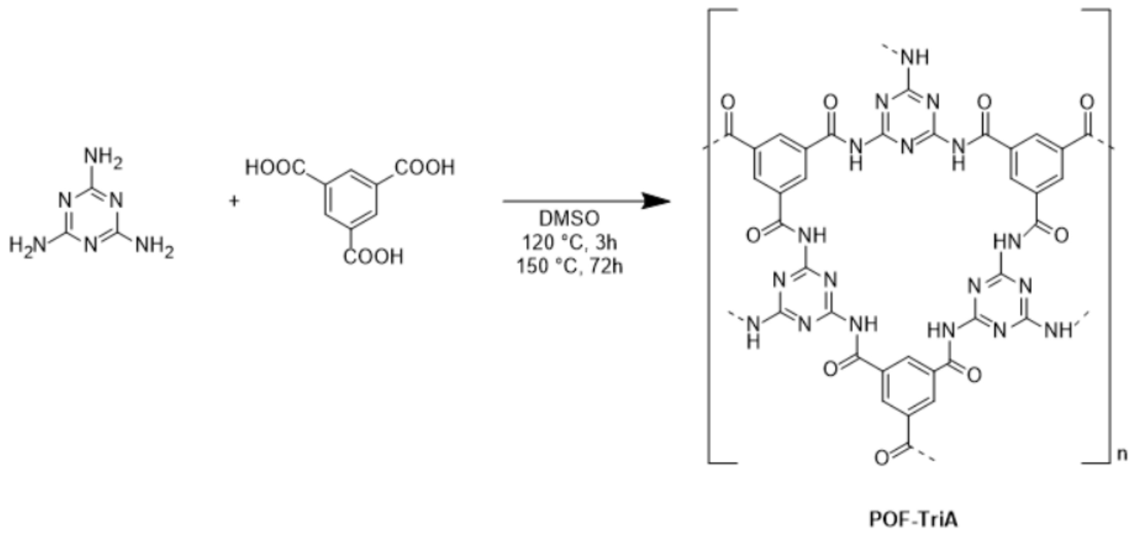

Scheme S3. Synthesis of POF-TriA.

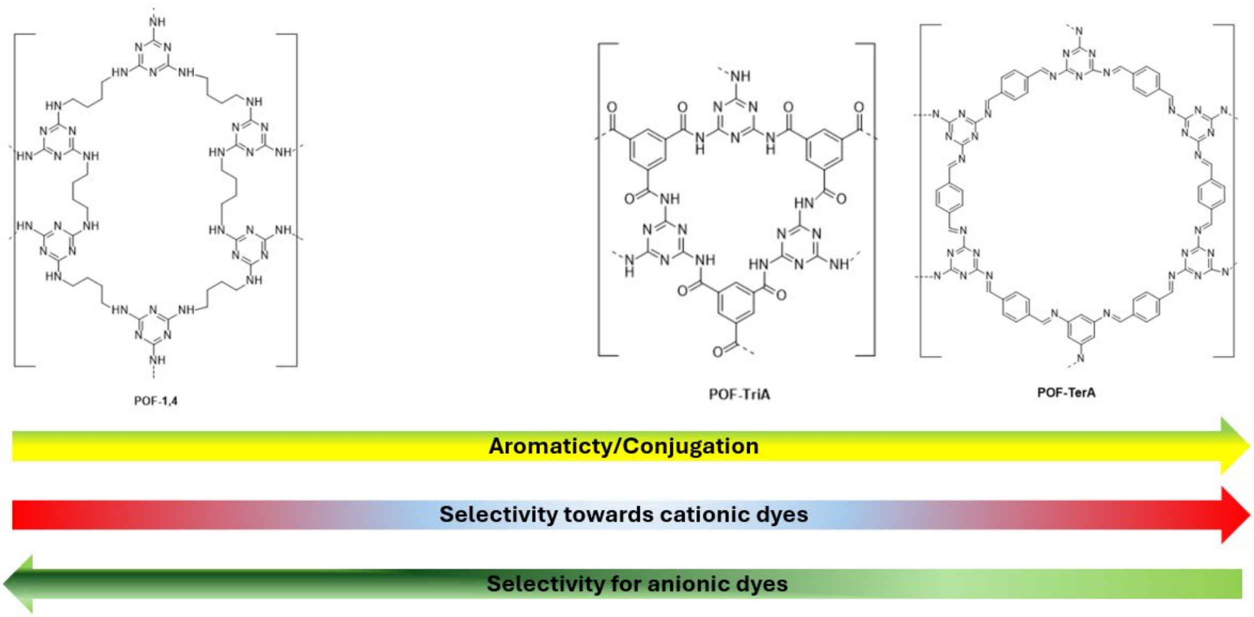

Scheme S4. Schematic summary of aromaticity-adsorption performance for POFs towards dyes.

Kinetic models

Adsorption kinetics were carried out putting in contact 7 mL of a dye solution (1.8 · 10^{-4} M) with 5 mg of POF, at 298 K. q_e values were obtained time by time and trends of q_e as a function of time were analyzed using both pseudo-first and pseudo-second order kinetic models, according to equations 1 and 2:

$$q_t = q_e \cdot \left[1 - e^{(-k_1 \cdot t)}\right] \text{(S1)}$$

$$q_t = \frac{k_2 \cdot {q_e}^2 \cdot t}{1 + k_2 \cdot q_e \cdot t} \text{. (S2)}$$

where q_t and q_e represent the adsorption capacity at a given time t and at the equilibrium, respectively, and k_1 and k_2 are pseudo-first and pseudo-second order kinetic constants.

Intra-particle diffusion model

The Intraparticle diffusion model was firstly describe by Weber–Morris.[4,5] This model is usually written as:

$q_t = k_{id}\, t^{1/2} + C$ (S3)

where q_t represents the amount adsorbed at time t, $k_{id}$is the intra-particle diffusion rate constant and C is intercept related to boundary layer thickness.

Isotherm binding models

To gain insights on the properties of the adsorbent materials and the mechanism of adsorption, the adsorption isotherms were determined. Different models have been developed, and in this case, we firstly considered POF-TriA, in the presence of all dyes, analyzing the results with different adsorption isotherms, among which the Langmuir[6] and Freundlich[7] ones gave rise to the high correlation coefficients and will be further discussed The Langmuir model is described by equation 4:

$$qe = \frac{K_L * q_m * C_e}{1 + K_L * C_e} \text{ (S4)}$$

where q_e and C_e are the adsorption capacity (mg/g) and the solute concentration (mg/L) at the equilibrium, K_L (L/mg) is the Langmuir constant that accounts for the affinity between the solute and the active sites on the adsorbent and q_m (mg/g) represents the maximum adsorption capacity. This model assumes homogeneous surfaces, that contain only one type of binding sites, so that the adsorption energy is constant.

On the other hand, the Freundlich model is described by equation 5:

$$q_e = K_F * C_e^{(1/n)} \quad (S5)$$

where K_F and n are empirical parameters representing the adsorption affinity and the heterogeneity of the adsorbent surface, respectively. In this case, the adsorption surface is considered heterogeneous, and the adsorption process gives rise to the formation of a multilayer. In this case, some adsorption sites are favored from an energetic point of view and the adsorption process generates an exponential decrease of the adsorption enthalpy.

Determination of adsorption thermodynamic parameters

To determine the thermodynamic parameters of the adsorption process, the distribution coefficient $K_d = (q_e/C_e)$ where q_e is expressed in mg/g and C_e in mg/L, was used as an approximation of the equilibrium constant, in assumption of quasi-linear regime under the conditions used. Before applying the van' t Hoff equation, K_d values, expressed in L/g, were converted into the dimensionless constant K^0, according to the following equation (6)

$$K^0 = K_d \cdot MW_{dye} \cdot C^0 \quad (S6)$$

where MW_{dye} is the molar mass of the dye, expressed in g/mol, and C^0 is the concentration of dye at the standard state, equal to 1 mol/L, assuming unitary activity coefficient in dilute solutions.
Subsequently, the values of $\ln(K^0)$ were plotted as a function of the inverse of temperature (1/T), and thermodynamic parameters, ΔH and ΔS, were determined by fitting of the plot by the van't Hoff equation (7)

$$\ln(K^0)_T = \frac{\Delta S}{R} - \frac{\Delta H}{RT} \quad (S7)$$

Table S1. q_e values determined in the presence of POF-TriA, as a function of volume of solutions of different dyes.

POF-TriA/MO	
V (L)	q_e (mg/g)[a]
0.001	12
0.002	23
0.003	35
0.004	47
0.007	81
0.010	110
0.013	152
0.017	161
0.020	175
**POF-TriA/EY**	
0.001	25
0.002	50
0.003	75
0.004	99
0.007	147
0.010	167
0.020	167
0.040	167
**POF-TriA/MB**	
0.001	9
0.002	18
0.003	23
0.004	25
0.010	27
0.020	28
0.060	28
**POF-TriA/RhB**	
0.001	17
0.002	34
0.003	51
0.004	68
0.007	118
0.010	156
0.013	186
0.020	206
0.030	218
0.080	226

[a] q_e were reproducible within ± 3%.

Table S2. q_e values determined in the presence of POF-TerA, as a function of volume of solutions of different dyes.

POF-TerA/MO	
V (L)	q_e (mg/g)[a]
0.001	12
0.002	24
0.003	36
0.004	47
0.007	74
0.010	83
0.013	84
0.017	112
0.020	114
**POF-TerA/RhB**	
0.001	18
0.002	35
0.003	53
0.004	70
0.007	124
0.010	152
0.013	198
0.017	236
0.020	289
0.030	360
0.040	381
0.060	408
0.080	429

[a] qe were reproducible within ± 3%.

Table S3. q_e values determined in the presence of POF-1,4, as a function of volume of solutions of different dyes.

V (L)	q_e (mg/g)[a]
**POF-1,4/MO**	
0.001	12
0.002	24
0.003	37
0.004	47
0.007	48
0.010	84
0.013	120
0.017	200
0.020	223
0.040	307
0.060	314
0.080	317
**POF-1,4/RhB**	
0.001	11
0.002	22
0.003	30
0.004	38
0.007	45
0.010	38
0.013	54
0.017	50
0.020	68
0.040	91
0.060	91
0.080	138

[a] q_e were reproducible within ± 3%.

Table S4. Intra-particle diffusion in adsorption all dyes as a function of different POFs.

Dye	k_{1ip} (mg/g·min)	C_1 (mg/g)	R^2	k_{2ip} (mg/g·min)	C_2 (mg/g)	R^2	k_{3ip} (mg/g·min)	C_3 (mg/g)	R^2
			POF-1,4						
MO	4.11 ± 0.07	(0.13 ± 0.60		2.12 ± 0.05	24.2 ± 0.9	0.992			
			POF-TerA						
MO	2.94 ± 0.13	1.96 ± 0.99	0.929	1.24 ± 0.08	20.8 ± 1.6	0.964			
RhB	2.84 ±0.05	-0.83 ± 0.48	0.989	0.77 ± 0.32	32.4 ± 6.6	0.372			
			POF-TriA						
MO	9.51 ± 0.83	6.17 ± 3.57	0.954	3.32 ± 0.11	39.8 ± 1	0.994	0.65 ± 0.05	68.8 ± 0.72	0.940
RhB	(6.86 ± 0.62)	17 ± 4	0.910	(2.02 ± 0.14	62.3 ± 1.9	0.941			

Table S5. Fitting parameters for the Freundlich model, as a function of POF and dye nature.

	K_F(mg^{1-n}L^n)/g	n	R^2
	POF-1,4		
MO	122 ± 14	3.5± 0.5	0.919
	POF-TerA		
RhB	80 ± 15	2.3 ±0.3	0.949
MO	37 ± 4	4.4 ±0.6	0.919
	POF-TriA		
RhB	71 ± 9	3.5 ± 0.5	0.927
MB	6 ± 1	2.6 ± 0.6	0.836
MO	128 ± 6	10 ± 2	0.834
EY	64 ± 12	4 ± 1	0.814

Table S6. Comparison among data collected using POF-1,4/MO and POF-Tera/RhB and data previously reported in literature.

Adsorbent	q_M(mg/g) MO	q_e (mg/g) RhB	Time	pH	Washing treatment	Reuse times	Reference
POF-TerA	329	472	7 h	6.7			This work
POF-1,4	431		7 h	6.7			This work
POF-TriA	175	233	7 h	6.7	Methanol wash	2-3	
CTF-1	256	469	55 min	3	Ethanol wash	4	[8]
BIM-COF			160 min	11	Aqueous NaBr	5	[9]
Ttba-TPDA-COF		833	10 min	2-11	Methanol wash	5	[10]
TS-COF-1	460	625	4 h	NA	Ethanol wash	3	[11]
S-iCOF	192.5		2 h	3	Aqueous $NaNO_3$ / Ethanol/HCl 5 M	3	[12]
BPM-COF	185		6 min	8	HCl 0.1 M	4	[9]
TAPT-HMIPA-COF			90 min	7	Ethanol wash	5	[13]
CuP-DMNDA-COF/Fe	329	378	1 h	NA	Methanol wash	5	[14]

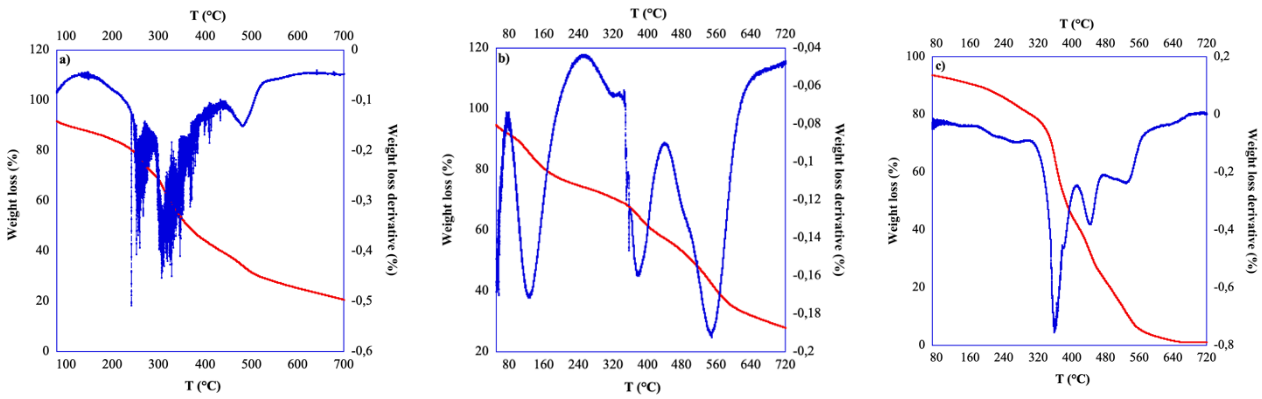

Figure S1. TGA and DTGA traces for: **a)** POF-1,4; **b)** POF-TerA; **c)** POF-TriA.

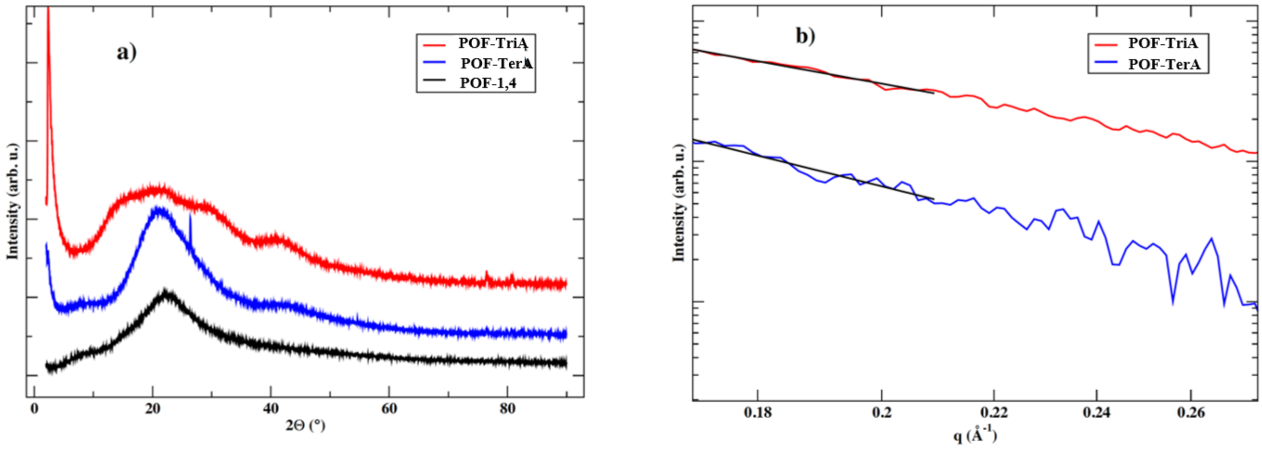

Figure S2. *a*) Measured PXRD patterns of POFs; *b*) Porod plot of POF-TFA and POF-TerA, with power-law regressions shown in black. Both axes are in logarithmic scale.

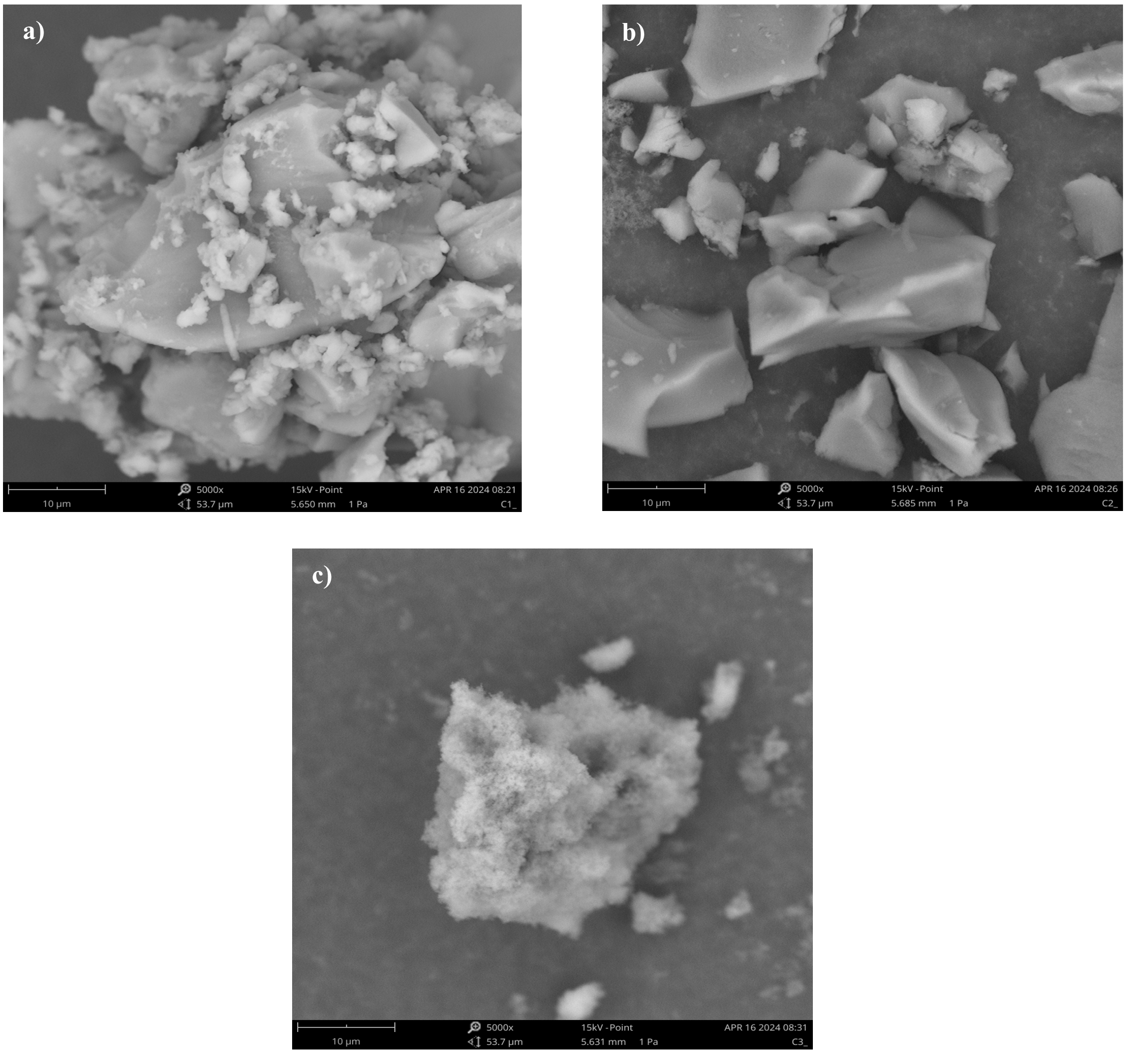

Figure S3. SEM images collected for: **a)** POF-1,4 (5000x); **b)** POF-TerA (5000X); **c)** POF-TriA (5000X).

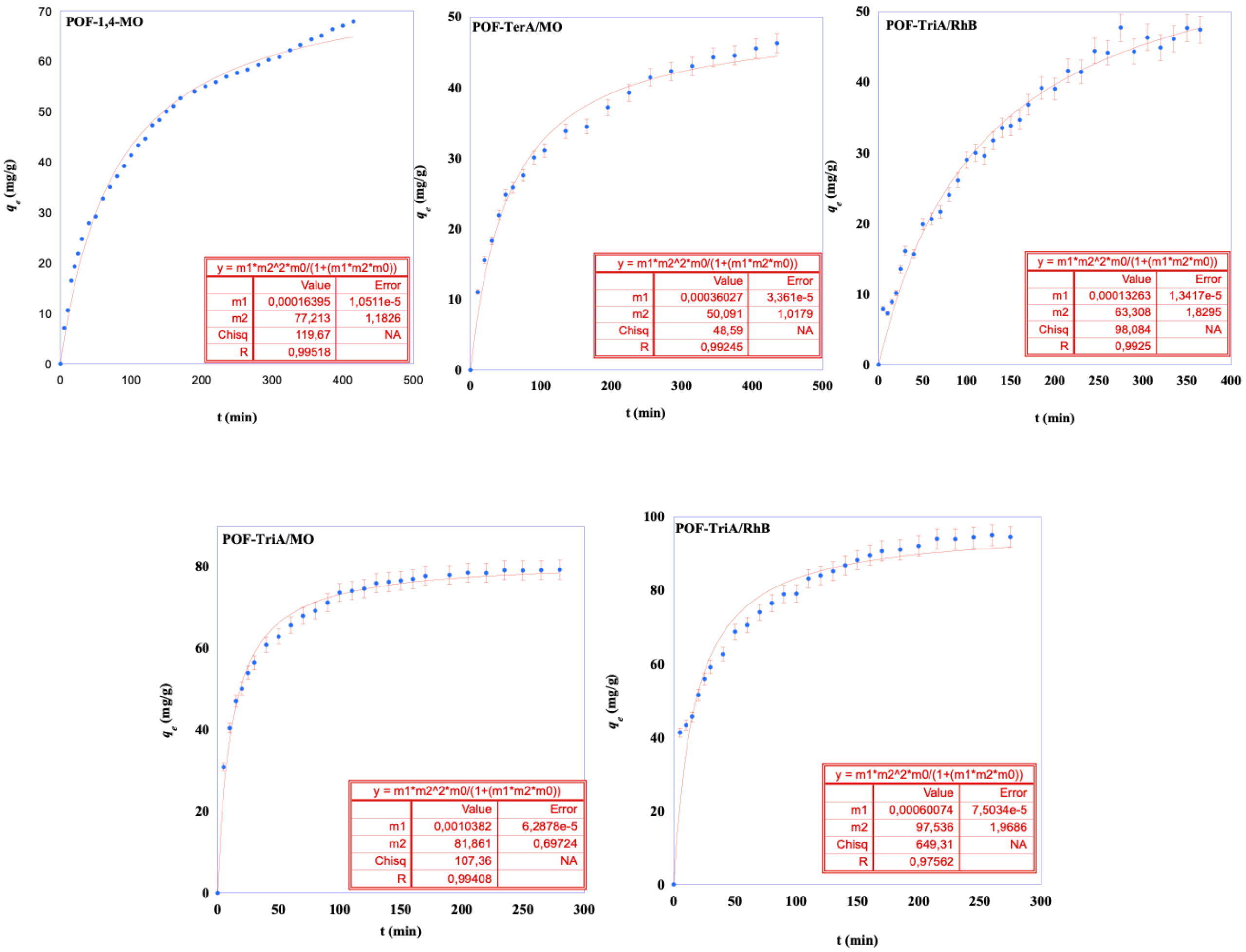

Figure S4. Plots of q_t values as a function of the time, at 298 K, for all POFs, in the presence of RhB or MO. Fitting according to the pseudo-second order kinetic model.

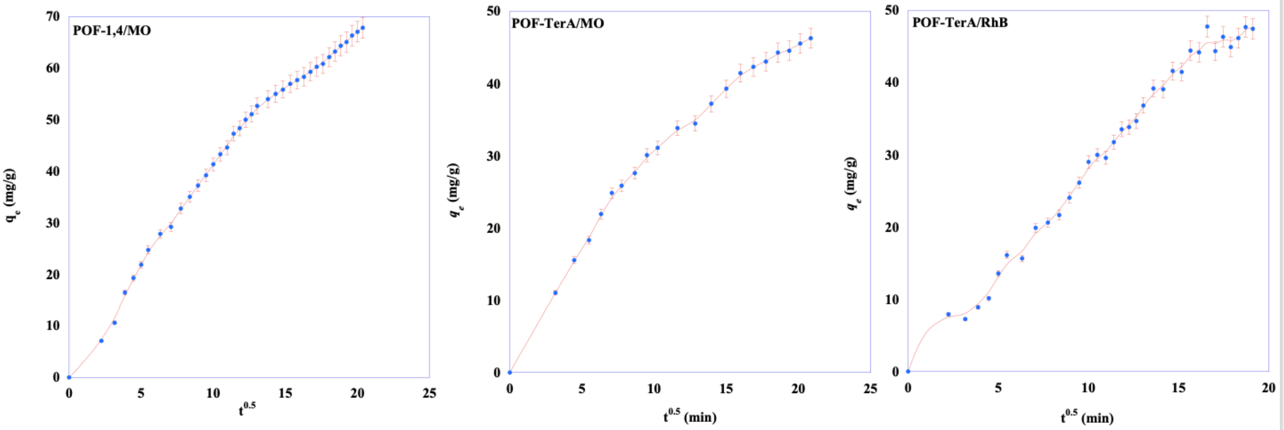

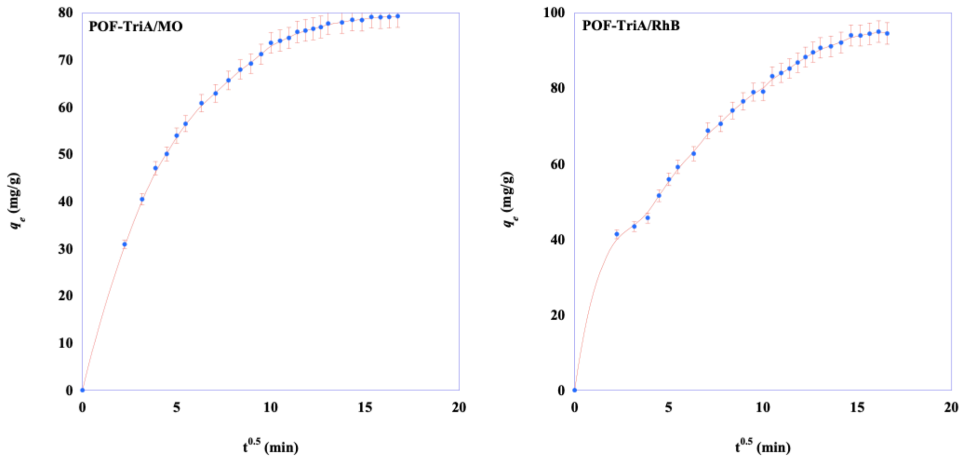

Figure S5. Intraparticle diffusion models for all POFs in the presence of MO or RhB at 1.8• 10^{-4} M.

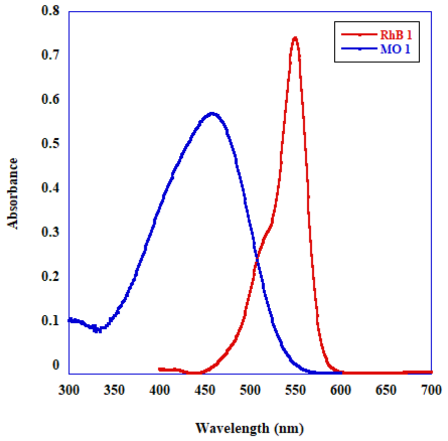

Figure S6. Superimposed adsorption spectra of MO and RhB.

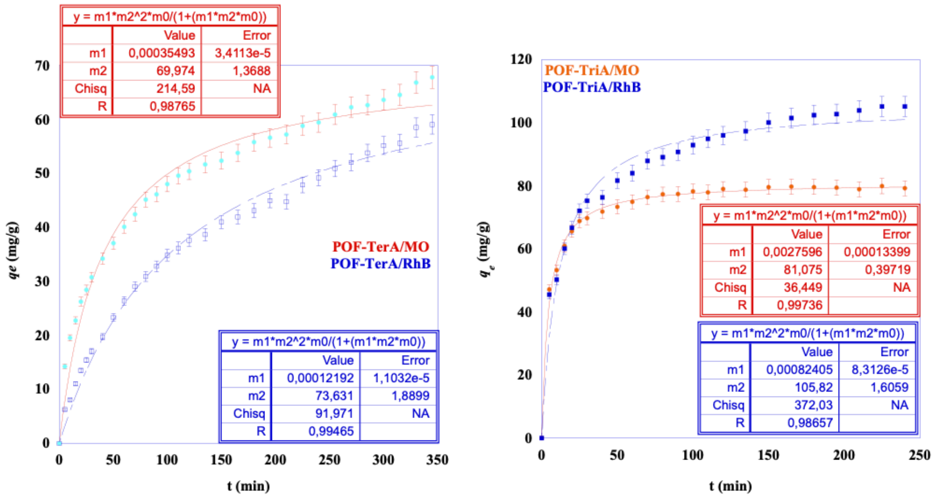

Figure S7. Plots of q_t values as a function of the time, at 298 K, for POF-TerA and POF-TriA, in the presence of mixtures of RhB or MO.

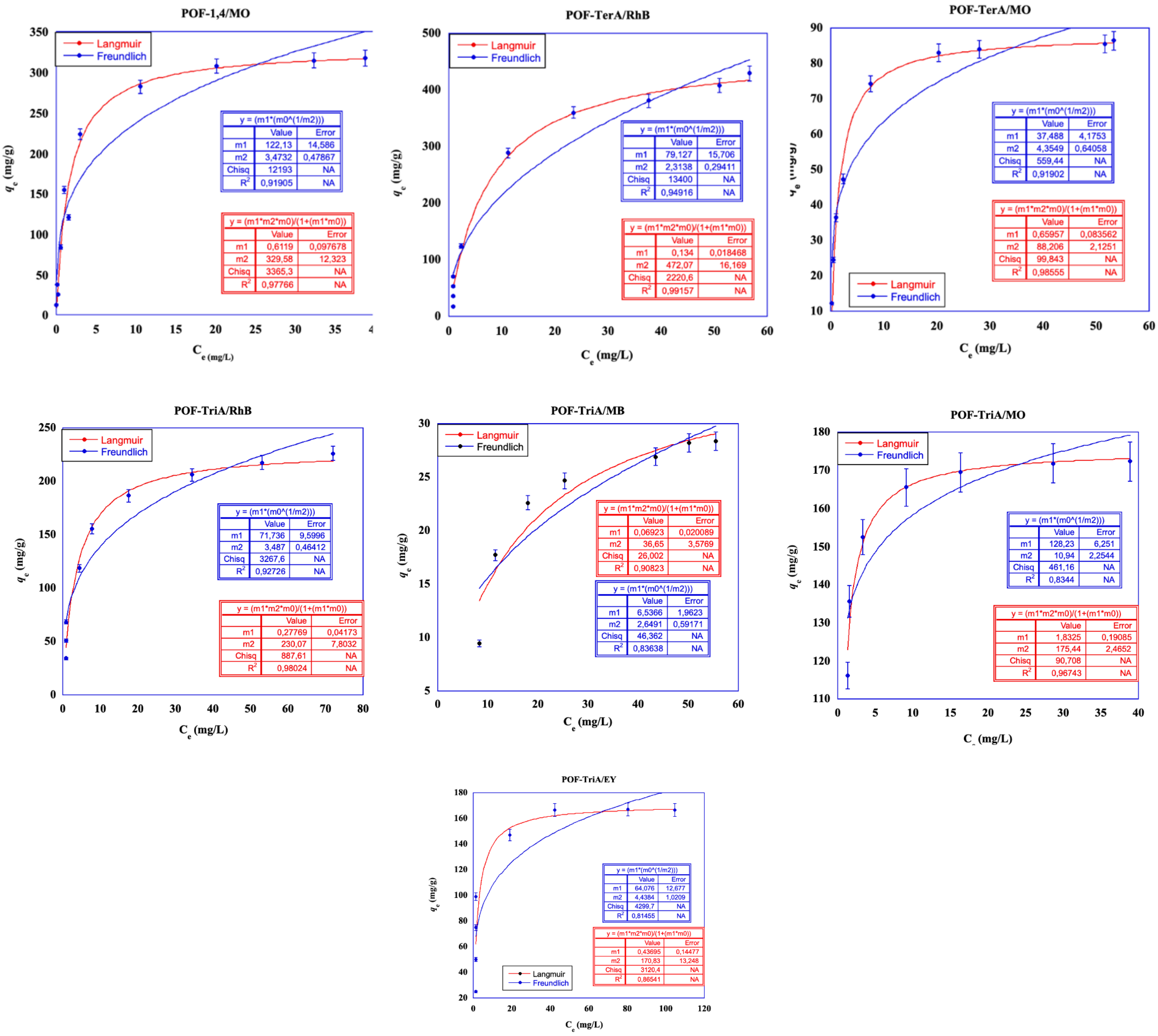

Figure S8. Plots of qe values as a function of C_e, at 298 K, for all POFs. q_e values were reproducible within ± 3%.

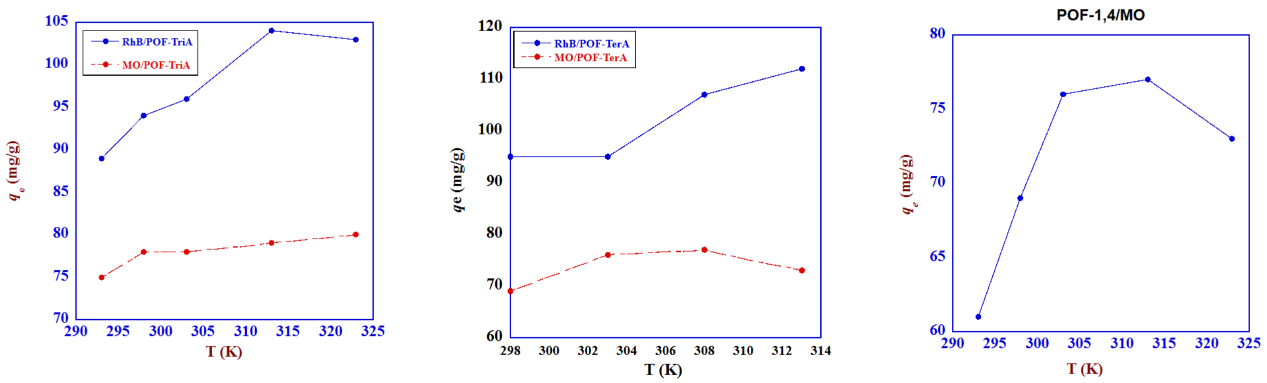

Figure S9. Plots of q_e values as a function of the temperatures for all POFs (5 mg), in the presence of RhB or MO solution ($1.8 \cdot 10^{-4}$ M; 20 mL). q_e values were reproducible within ± 3%.

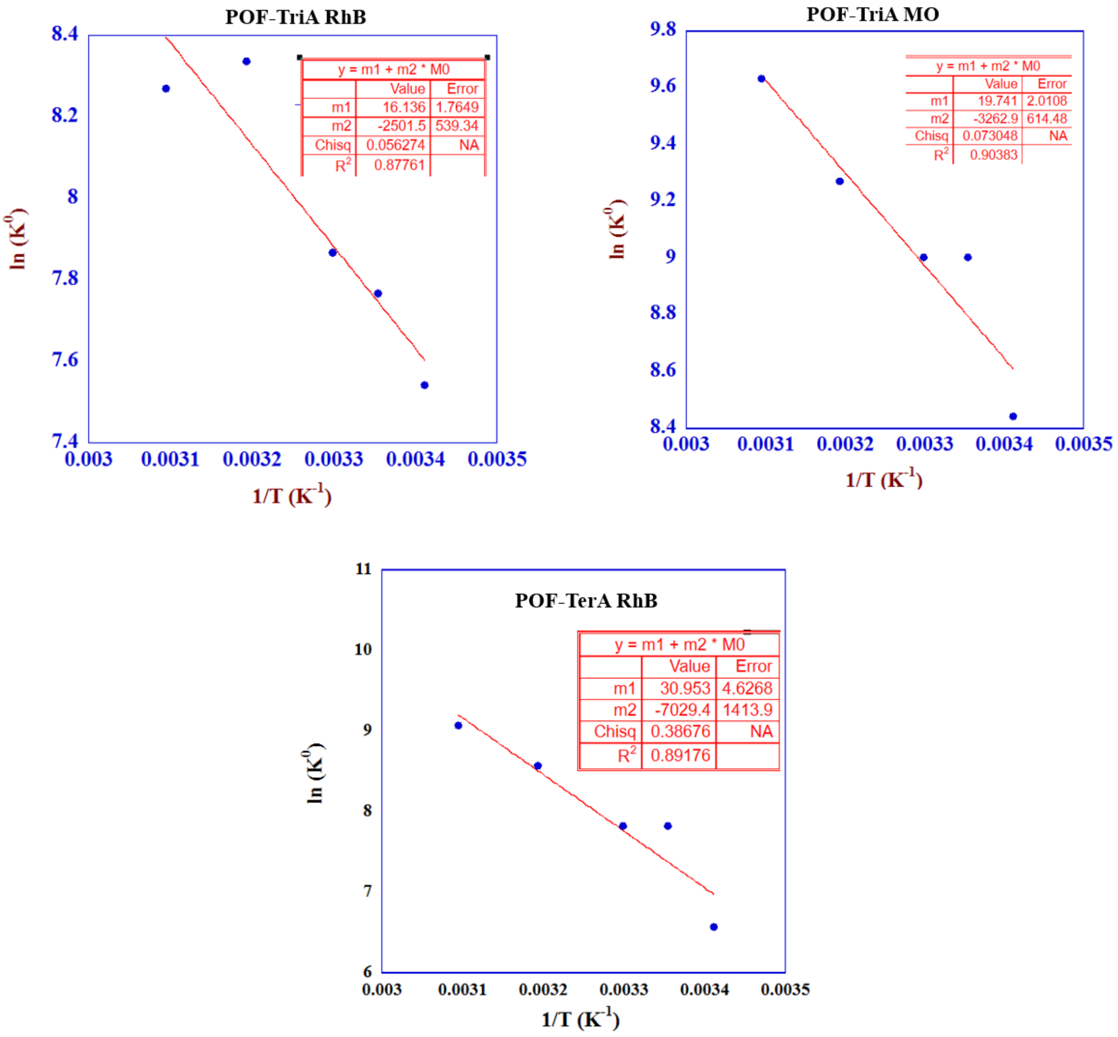

Figure S10. Plots of ln (q_e/C_e) as a function of 1/T for all POFs (5 mg), in the presence of RhB or MO solution (1.8 · 10^{-4} M; 20 mL).

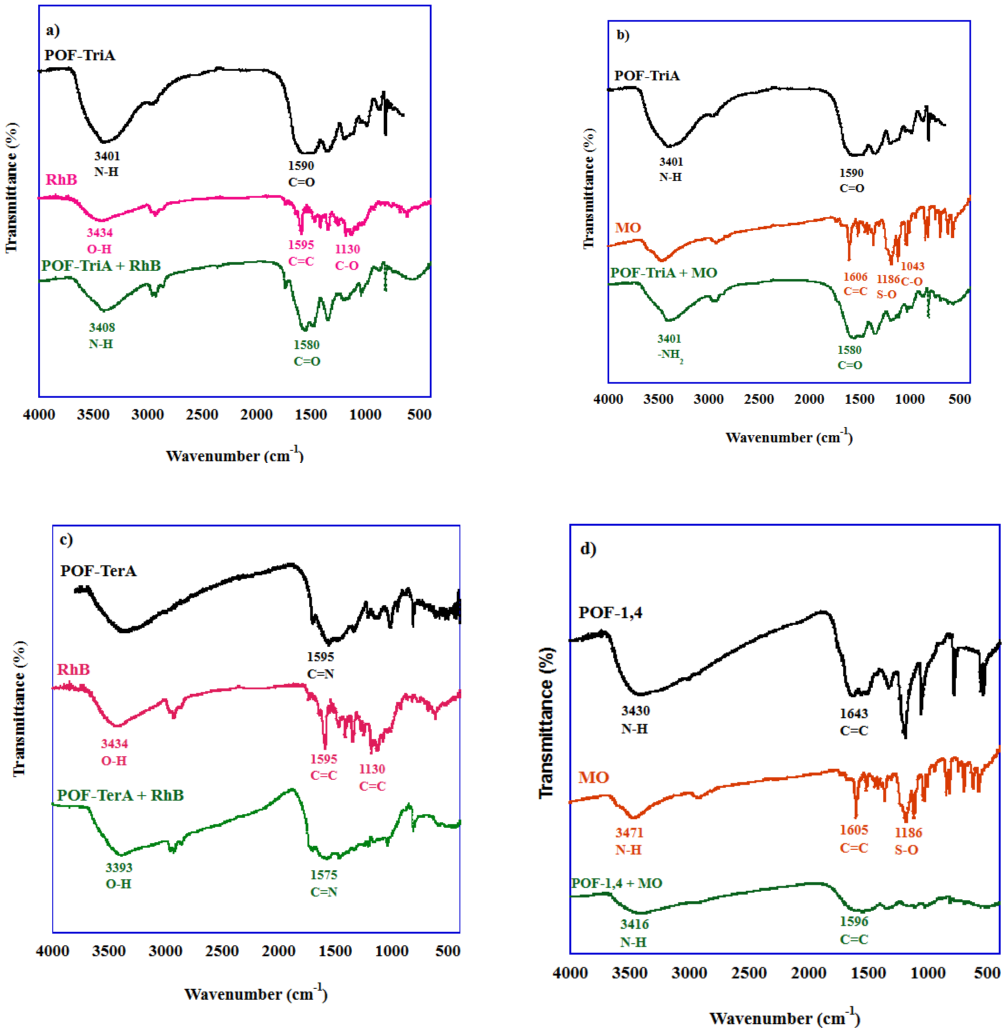

Figure S11. Stacked IR spectra of POFs upon adsorption of dyes and pristine materials.

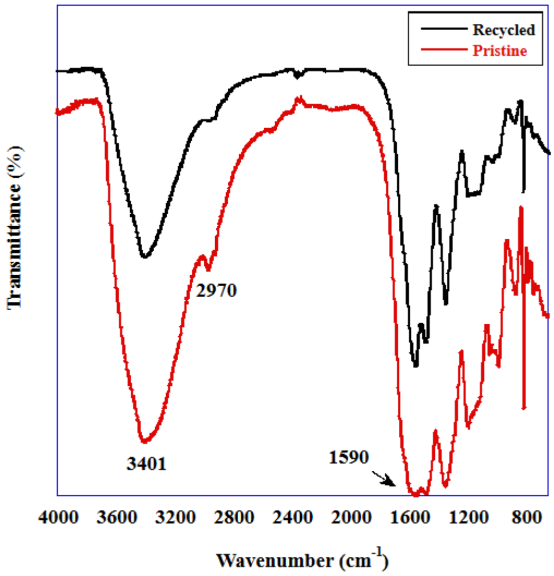

Figure S**12**. Stacked spectra of pristine and recycled POF-TriA.

References

1. Sahiner, N.; Demirci, S.; Sel, K. Covalent Organic Framework Based on Melamine and Dibromoalkanes for Versatile Use. *Journal of Porous Materials* **2016**, *23*, 1025–1035, doi:10.1007/s10934-016-0160-9.
2. Hamdi, F.; Roushani, M.; Nasibipour, M.; Hoseini, S.J. Aptasensor Based on High Surface Area Covalent Organic Framework for Simple and Ultrasensitive Detection of Sarcosine in the Diagnosis of Prostate Cancer. *Anal. Chim. Acta* **2024**, *1291*, 342235, doi:10.1016/j.aca.2024.342235.
3. Sarabaegi, M.; Roushani, M.; Hosseini, H.; Hoseini, S.J.; Bahrami, M. Facile Synthesis of a Covalent Organic Framework (COF) Based on the Reaction of Melamine and Trimesic Acid Incorporated Electrospun Nanofiber and Its Application as an Electrochemical Tyrosinamide Aptasensor. *New Journal of Chemistry* **2020**, *44*, 14922–14927, doi:10.1039/D0NJ02837A.
4. Ahmed, M.J.; Theydan, S.K. Microwave Assisted Preparation of Microporous Activated Carbon from Siris Seed Pods for Adsorption of Metronidazole Antibiotic. *Chemical Engineering Journal* **2013**, *214*, 310–318, doi:10.1016/j.cej.2012.10.101.
5. Wang, J.; Guo, X. Adsorption Isotherm Models: Classification, Physical Meaning, Application and Solving Method. *Chemosphere* **2020**, *258*, 127279, doi:10.1016/j.chemosphere.2020.127279.
6. Choy, K.K.H.; Porter, J.F.; McKay, G. Langmuir Isotherm Models Applied to the Multicomponent Sorption of Acid Dyes from Effluent onto Activated Carbon. *J. Chem. Eng. Data* **2000**, *45*, 575–584, doi:10.1021/je9902894.
7. Shulepov, S.Yu. A Dynamic Approach to Brownian Coagulation of Electrostatically Stabilized Colloid Particles. Diffusion Relaxation of the Adsorption Layer. *J. Colloid Interface Sci.* **1997**, *189*, 199–207, doi:10.1006/jcis.1997.4813.
8. Wang, T.; Kailasam, K.; Xiao, P.; Chen, G.; Chen, L.; Wang, L.; Li, J.; Zhu, J. Adsorption Removal of Organic Dyes on Covalent Triazine Framework (CTF). *Microporous and Mesoporous Materials* **2014**, *187*, 63–70, doi:10.1016/j.micromeso.2013.12.016.
9. Rahimi, J.; Ijdani, M.T.; Abbasi, H.; Salehi, M.M.; Maleki, A. Two-Dimensional Imide-Based Covalent Organic Frameworks for Cationic Dye Adsorption: Synthesis, Characterization, Isotherm, Kinetics, and Thermodynamic Analysis. *Journal of Hazardous Materials Advances* **2025**, *18*, 100680, doi:10.1016/j.hazadv.2025.100680.
10. Xu, S.-X.; Yao, Z.-Q.; Zhang, Y.-H. A Covalent Organic Framework Exhibiting Amphiphilic Selective Adsorption toward Ionic Organic Dyes Tuned by PH Value. *Eur. Polym. J.* **2020**, *133*, 109764, doi:10.1016/j.eurpolymj.2020.109764.
11. Zhu, X.; An, S.; Liu, Y.; Hu, J.; Liu, H.; Tian, C.; Dai, S.; Yang, X.; Wang, H.; Abney, C.W.; et al. Efficient Removal of Organic Dye Pollutants Using Covalent Organic Frameworks. *AIChE Journal* **2017**, *63*, 3470–3478, doi:10.1002/aic.15699.
12. Lu, S.; Zhang, K.; Wu, Y.; Duan, F.; Du, M. Facile Synthesis of Cationic Covalent Organic Frameworks with Abundant Protonated Pyridine Nitrogen Groups for Selective Absorption of Organic Dyes. *New Journal of Chemistry* **2025**, *49*, 3946–3955, doi:10.1039/D4NJ05381H.
13. Rastegari, F.; Asghari, S.; Mohammadpoor-Baltork, I.; Sabzyan, H.; Tangestaninejad, S.; Moghadam, M.; Mirkhani, V. A PH-Dependent and Charge Selective Covalent Organic Framework for Removal of Dyes from Aqueous Solutions. *J. Hazard. Mater.* **2024**, *476*, 135075, doi:10.1016/j.jhazmat.2024.135075.
14. Hou, Y.; Zhang, X.; Wang, C.; Qi, D.; Gu, Y.; Wang, Z.; Jiang, J. Novel Imine-Linked Porphyrin Covalent Organic Frameworks with Good Adsorption Removing Properties of RhB. *New Journal of Chemistry* **2017**, *41*, 6145–6151, doi:10.1039/C7NJ00424A.
